# Reduced hepatic bradykinin degradation accounts for cold-induced BAT thermogenesis and WAT browning in male mice

**DOI:** 10.1038/s41467-023-38141-0

**Published:** 2023-05-02

**Authors:** Fei Xiao, Haizhou Jiang, Zi Li, Xiaoxue Jiang, Shanghai Chen, Yuguo Niu, Hanrui Yin, Yousheng Shu, Bo Peng, Wei Lu, Xiaoying Li, Zhigang Li, Shujue Lan, Xiaoyan Xu, Feifan Guo

**Affiliations:** 1 Zhongshan Hospital, Institute for Translational Brain Research, State Key Laboratory of Medical Neurobiology, MOE Frontiers Center for Brain Science, Fudan University, Shanghai, China; 2 CAS Key Laboratory of Nutrition, Metabolism and Food Safety, Shanghai Institute of Nutrition and Health, University of Chinese Academy of Sciences, Chinese Academy of Sciences, Shanghai, China; 3 Center for Excellence in Molecular Cell Science, Chinese Academy of Sciences, Shanghai, China; 4 Core Facility Center, CAS Center for Excellence in Molecular Plant Sciences, Chinese Academy of Sciences, Shanghai, China

**Keywords:** Endocrine system and metabolic diseases, Metabolism

## Abstract

An important role for liver in the regulation of adipose tissue thermogenesis upon cold exposure has been suggested; however, the underlying mechanisms remain incompletely defined. Here, we identify elevated serum bradykinin levels in response to acute cold exposure in male mice. A bolus of anti-bradykinin antibodies reduces body temperature during acute cold exposure, whereas bradykinin has the opposite effect. We demonstrate that bradykinin induces brown adipose tissue thermogenesis and white adipose tissue browning, and bradykinin increases uncoupling protein 1 (UCP1) expression in adipose tissue. The bradykinin B2 receptor (B2R), adrenergic signaling and nitric oxide signaling are involved in regulating bradykinin-increased UCP1 expression. Moreover, acute cold exposure inhibits hepatic prolyl endopeptidase (PREP) activity, causing reduced liver bradykinin degradation and increased serum bradykinin levels. Finally, by blocking the breakdown of bradykinin, angiotensin-converting enzyme inhibitors (ACEIs) increase serum bradykinin levels and induce brown adipose tissue thermogenesis and white adipose tissue browning via B2R. Collectively, our data provide new insights into the mechanisms underlying organ crosstalk in whole-body physiology control during cold exposure and also suggest bradykinin as a possible anti-obesity target.

## Abstract (web summary)

Adipose tissue thermogenesis plays a role in appropriate response to cold exposure through incompletely understood mechanisms. Here the authors report that acute cold exposure increases the serum levels of bradykinin, which induces brown adipose tissue thermogenesis and white adipose tissue browning in male mice.

## Introduction

The ability to respond appropriately to cold exposure and maintain a constant body temperature regardless of the ambient temperature is essential for the survival of homeothermic vertebrates. Adipose tissue plays an important role in protection against the cold through non-shivering thermogenesis (NST)^1^. Generally, adipocytes can be categorized as white, brown and beige fat cells^2^. Some brown fat-like cells may appear in the white adipose tissue (WAT) following external stimulation. These cells are known as “beige cells” and this process is known as white fat browning^2^. Both brown and beige fat cells express the unique uncoupling protein 1 (UCP1), which is involved in heat generation by uncoupling mitochondrial fatty acid oxidation from the production of ATP^1^.

Cold exposure enhances brown adipose tissue (BAT) activity and induces WAT browning to maintain body temperature. These processes are mainly controlled by the sympathetic nervous system (SNS) via noradrenaline^3^. Meanwhile, multiple signals from peripheral tissues also regulate these processes^3^. Recent data suggest that the liver contributes to thermogenesis through secreted factors or neural signals^4–10^. For example, liver-derived acylcarnitines serve as fuel sources for BAT thermogenesis during cold exposure^4^. Activin E is a liver-secreted factor that regulates BAT activity and WAT browning^6^. The hepatokine tsukushi (TSK) has also been suggested to affect thermogenesis in brown fat^5^, but these results still need to be confirmed^11^. Fibroblast growth factor 21 (FGF21) can promote brown and beige fat thermogenesis^7^, and liver-derived FGF21 maintains core body temperature during cold exposure^8^. Hepatic glucokinase and c-Jun affect BAT thermogenesis via the vagus nerve^9,10^. However, the crosstalk between liver and adipose tissue remains largely unknown, and more research is needed to identify additional mechanisms that drive thermogenesis during cold challenge.

In recent years, there has been an increase in the global prevalence of metabolic syndrome including obesity. Obesity results from an energy imbalance, a situation where energy intake exceeds consumption^12^. As methods to dissipate energy, stimulating BAT activity and inducing WAT browning have been considered appealing therapeutic strategies to combat obesity and related metabolic diseases^1,12^. The identification of novel circulating factors that may work as cold mimetics to promote energy expenditure is essential to uncover new therapeutic targets for obesity.

Bradykinin (BK) is a circulating peptide hormone and a principal effector of the kallikrein-kinin system (KKS), an endogenous proteolytic system that when triggered results in the release of kinins from kininogens^13^. Kinins include BK and kallidin/ kallidin-like peptide (Lys-BK in humans/KLP or Arg-BK in rodents), and their metabolites des-Arg^9^-BK and des-Arg^10^-kallidin (des-Arg^10^-KLP in rodents)^13^. BK is derived from high-molecular-weight kininogen (HMWK) and acts via the bradykinin receptor B2 (B2R). B2R is a member of the seven-transmembrane G-protein-coupled receptor superfamily and is constitutively expressed in a wide variety of tissues^14^. BK is implicated in many physiological and pathological states, including vasodilation, inflammation, and pain^15^. Limited studies also suggest that the BK is involved in body temperature maintenance. For example, intracerebroventricular (i.c.v.) injection of BK has been reported to promote core temperature^16^. Considering these studies, the role of BK in adipose tissue thermogenesis and body temperature during cold exposure remains elusive.

In the present study, we identified elevated levels of serum BK in response to acute cold stress. A bolus of anti-BK antibodies reduced body temperature during acute cold, whereas BK had the opposite effect. Further studies demonstrated that BK increased UCP1 expression, elevated BAT thermogenesis, and promoted WAT browning. These effects were attenuated in adipose tissue-specific B2R knockout mice. In addition, cold-induced free fatty acids inhibited hepatic prolyl endopeptidase (PREP) activity to protect BK from degradation and thus increased hepatic and serum BK levels. By blocking BK breakdown, the angiotensin-converting enzyme inhibitors (ACEIs) could increase serum BK levels and had similar effects on adipose tissue as BK. Our study provides new insights into crosstalk among different tissues in the regulation of body temperature. These observations also uncover a function of BK in thermogenesis and suggest it as a potential target for therapeutic intervention in obesity.

## Results

### Serum bradykinin levels are increased and BK is required to sustain the body temperature during acute cold

**Fig. 1:**
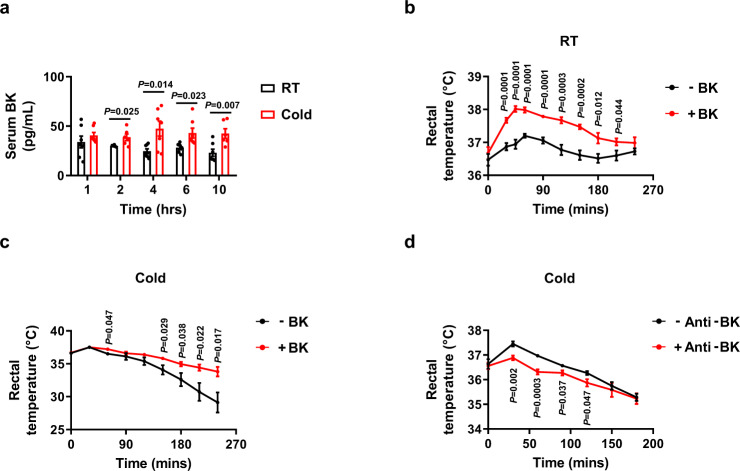
Serum bradykinin levels are increased and bradykinin is required to sustain the body temperature during acute cold exposure. **a** Serum BK levels of male WT mice exposed to 25 °C (RT) or 4 °C (Cold) for different amounts of time in the absence of food and water. **b** Rectal temperature in male WT mice i.p. injected with a single dose of PBS (–BK) or 1 mg/kg BK ( + BK) at 25 °C (RT) in the absence of food and water. **c** Rectal temperature during acute cold tolerance test in male WT mice i.p. injected with a single dose of PBS (–BK) or 1 mg/kg BK (+BK). **d** Rectal temperature during acute cold tolerance test in male WT mice i.p. injected with a single dose of 10 μg/mouse IgG (–Anti-BK) or anti-BK ( + Anti-BK) antibodies. For **c** and **d**, the mice were exposed to cold immediately after injection in the absence of food and water. For **a**, *n* = 7 (RT 1 h, 4 h, 10 h; Cold 1 h, 4 h, 6 h) and 6 (RT 2 h, 6 h; Cold 2 h, 10 h) mice per group. For **b**, *n* = 7 in each group. For **c**, *n* = 5 (–BK) and 6 (+BK) mice per group. For **d**, *n* = 6 (–Anti-BK) and 7 (+Anti-BK) mice per group. Mean ± SEM are representative of at least two independent experiments (**a–d**); two-tailed unpaired Student’s *t*-test (**a**), or two-way RM ANOVA with Geisser-Greenhouse’s correction followed by post hoc unpaired *t*-test (**b-d**). Source data are provided as a Source data file.

To elucidate the role of BK during acute cold exposure, mice were placed at room temperature (25 °C) or under cold conditions (4 °C) for different amounts of time, and the serum levels of BK as well as the other three kinins including des-Arg^9^-BK, kallidin-like peptide (KLP, Arg-BK) and des-Arg^10^-KLP were measured. We found that serum BK levels of mice under cold exposure were higher than those maintained at room temperature at each time point (examined as early as 2 h and as late as 10 h, Fig. 1a, S1a). Consistently, the levels of its metabolite, des-Arg^9^-BK, were also increased in response to cold exposure at these time points (Fig. S1b). In contrast, the levels of the other two kinins were either increased at the very late time point (10 h) or remained unchanged following cold exposure (Fig. S1c, S1d), suggesting that they are unlikely to contribute to the reduced body temperature under conditions of acute cold exposure.

To assess the role of BK in the control of body temperature, we first injected wild-type (WT) mice with phosphate-buffered saline (PBS) or 1 mg/kg BK^17^ at room temperature. We observed much higher serum BK levels in the BK-treated mice than that in the control mice 5 min after injection (Fig. S2a). We found that an intraperitoneal (i.p.) injection of BK acutely increased the body temperature at room temperature compared with control treatment (Fig. 1b). Moreover, BK improved the ability to maintain body temperature following acute cold exposure (Fig. 1c). To further confirm the role of BK during acute cold exposure, we investigated whether anti-BK antibodies hampered the ability to maintain body temperature during acute cold exposure. As predicted, injection with a single dose of 10 μg anti-BK antibodies per mouse accelerated the reduction in body temperature during acute cold exposure (Fig. 1d).

As mice and humans age, loss of BAT function and increased sensitivity to cold are observed^18^. Therefore, we compared serum BK levels between 10-week-old and 18-month-old mice. We found that serum BK levels were elevated in response to acute cold exposure in 10-week-old mice, whereas no change was observed in 18-month-old mice (Fig. S3a). Notably, older mice exhibited higher basal BK levels at room temperature (Fig. S3a). Furthermore, BK increased the body temperature at room temperature in aged mice (Fig. S3b) and reversed cold sensitivity in aged mice placed at 4 °C (Fig. S3c).

### Bradykinin increases uncoupling protein 1 (UCP1) expression in BAT and WAT

**Fig. 2:**
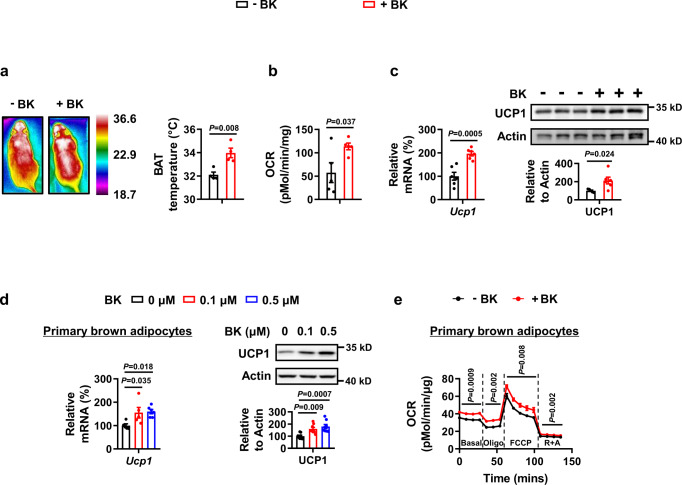
Bradykinin increases uncoupling protein 1 (UCP1) expression in brown adipose tissue. **a–c** Infrared images (**a**), oxygen consumption rate (OCR, **b**)  *Ucp1* mRNA (**c,** **left**) and protein levels (**c,** **right**) of BAT from male WT mice i.p. injected with a single dose of PBS (–BK) or 1 mg/kg BK ( + BK) for 30 mins in the absence of food and water at 25 °C. **d** *Ucp1* mRNA (left) and protein levels (right) of primary brown adipocytes treated with 0, 0.1 or 0.5 μM BK for 48 h. **e** OCR of primary brown adipocytes treated without (–BK) or with (+BK) 0.1 μM BK for 24 h. Reagents oligomycin (Oligo), carbonyl cyanide 4-(trifluoromethoxy) phenylhydrazone (FCCP), rotenone (R) and antimycin A (A) were added sequentially as indicated. For **a**, *n* = 4 in each group. For **b**, *n* = 5 in each group. For **c**, *n* = 6 in each group (left); *n* = 5 (–BK) and 6 (+BK) mice per group (right). For **d** *n* = 5 (0, 0.1 μM BK) and 6 (0.5 μM BK) in each group (left); *n* = 8 in each group (right). For **e**, *n* = 5 (–BK) and 6 (+BK) in each group. Mean ± SEM are representative of at least two independent experiments (**a–c**) or at least three independent experiments (**d,** **e**); two-tailed unpaired Student’s *t*-test (**a,** **b,** **c**), ordinary one-way ANOVA with Dunnett’s test (**d**), two-way RM ANOVA with Geisser-Greenhouse’s correction (**e**). Source data are provided as a Source data file.

Adaptive changes are required to maintain the body temperature in response to cold exposure; one such change is increased thermogenesis in BAT and browning in WAT^1,3,19^. Therefore, we next examined the effects of 1 mg/kg BK on BAT after a single injection. Infrared image analysis and oxygen consumption rate (OCR) measurement revealed that thermogenesis was higher in the BAT of mice treated with BK compared with that in the control group at room temperature (Fig. 2a, b) and under acute cold exposure (Fig. S4a, b). The mice rolled themselves up into a ball following acute cold exposure (Fig. S4a). The whole-body oxygen consumption was also increased in BK-treated mice compared with that in control mice at room temperature (Fig. S4d). UCP1 expression in adipose tissue is responsible for heat production and we investigated the effects of BK on UCP1 expression. Consistently, UCP1 was upregulated in the BAT of BK-treated mice at room temperature and during acute cold exposure (Fig. 2c, S4c). The direct effects of BK on brown adipose tissue were further tested by treatment of primary brown adipocytes with BK or control vehicle. As observed in vivo, UCP1 expression and OCR were also increased by BK in vitro (Fig. 2d, e, S4e, S2b).

Additionally, we examined whether WAT browning was induced following a single injection of 1 mg/kg BK. We examined the expression of genes related to WAT browning, including *peroxisome proliferative activated receptor gamma co-activator 1 alpha* (*Ppargc1α*), *cell death-inducing DNA fragmentation factor, alpha subunit-like effector A* (*Cidea*), *peroxisome proliferator activated receptor alpha (Ppara), cytochrome c oxidase subunit 7A1* (*Cox7a1*), *cytochrome c oxidase subunit 8B* (*Cox8b*) and *elongation of very long chain fatty acids (FEN1/Elo2, SUR4/Elo3, yeast)-like 3* (*Elovl3*)^20,21^. The mRNA abundance of these genes was not altered by a single injection of 1 mg/kg BK, either at room temperature or during acute cold exposure (Fig. S4f, S4g).

**Fig. 3:**
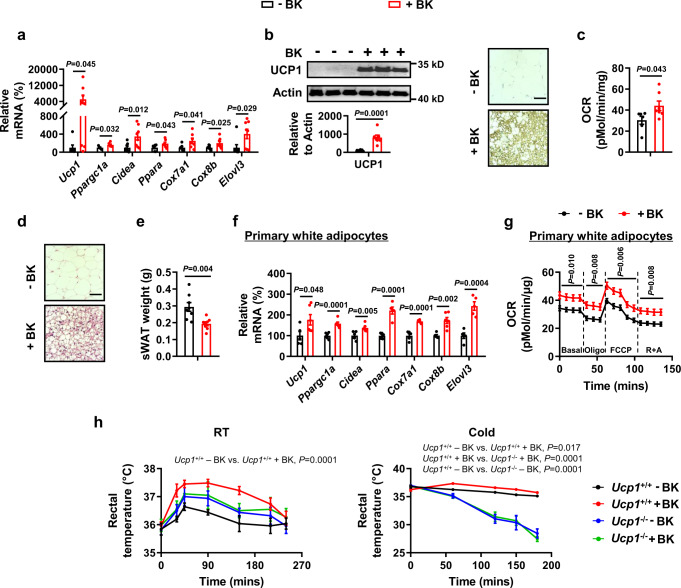
Bradykinin increases uncoupling protein 1 (UCP1) expression in white adipose tissue. **a–e** mRNA levels of browning marker genes in sWAT (**a**), UCP1 protein levels in sWAT (**b left**), representative immunohistochemistry (IHC) staining of UCP1 in sWAT (**b** **right**), OCR in sWAT (**c**), representative H&E staining of sWAT (**d**), and sWAT weight (**e**) from male WT mice i.p. injected with PBS (–BK) or 1 mg/kg BK ( + BK) once a day for 14 days at 25 °C. Scar bars, 50 μm (**b,** **d**). **f**, **g** mRNA levels of browning marker genes (**f**) and OCR (**g**) of primary white adipocytes treated without (–BK) or with (+ BK) 1 μM BK for 48 h. **h** Rectal temperature at 25 °C (RT) or 4 °C (Cold) in male control (*Ucp1*^+/+^) or *Ucp1* knockout (*Ucp1*^-/-^) mice i.p. injected with a single dose of PBS (–BK) or 1 mg/kg BK ( + BK) for different time. The mice were maintained at RT or exposed to cold immediately after injection in the absence of food and water. For **a**, *n* = 5-8 (–BK) or 6-8 (+BK) mice per group. For **b**, *n* = 8 (–BK) and 7 (+BK) in each group. For **c**, *n* = 6 (–BK) and 7 (+BK) in each group. For **e**, *n* = 8 in each group. For **f**, *n* = 5 or 6 in each group. For **g**, *n* = 6 in each group. For **h** *n* = 5 (*Ucp1*^+/+^–BK, *Ucp1*^-/-^–BK), 6 (*Ucp1*^+/+^+BK) and 4 (*Ucp1*^-/-^+BK) mice per group (left); *n* = 5 (*Ucp1*^+/+^–BK) and 6 (*Ucp1*^+/+^+BK, *Ucp1*^-/-^–BK, *Ucp1*^-/-^+BK) mice per group (right). Mean ± SEM are representative of at least two independent experiments (**a–e,** **h**) or at least three independent experiments (**f,** **g**); two-tailed unpaired Student’s *t*-test (**a,** **b,** **c,** **e,** **f**), two-way RM ANOVA with Geisser-Greenhouse’s correction (**g**), two-way RM ANOVA with Geisser-Greenhouse’s correction followed by Tukey’s multiple comparisons test (**h**). Source data are provided as a Source data file.

Probably, inducing WAT browning requires long-term treatment; therefore, we tested the effects of long-term BK treatment on UCP1 expression in WAT and BAT after injecting mice with 1 mg/kg BK once a day for 14 days at room temperature. In these experiments, we obtained different results from those observed immediately after a single BK injection; the expression levels of WAT browning markers, including *Ucp1*, *Ppargc1α*, *Cidea*, *Ppara*, *Cox7a1*, *Cox8b* and *Elovl3*, were increased in the subcutaneous WAT (sWAT) of these mice (Fig.3a, b). As expected, the OCR was increased in the sWAT of BK-treated mice (Fig.3c). BK decreased the adipocyte size and weight of the sWAT compared with those in control mice (Fig. 3d, e). The mRNA levels of browning markers and OCR were increased in primary white adipocytes treated with BK (Fig. 3f, g, S5a). We also assessed other physiological parameters in mice treated with 1 mg/kg BK once a day for 14 days at room temperature (Fig. S5b–j). Although the body weight was slightly reduced in BK-treated mice following injection for 14 days, the difference between the two groups was not statistically significant (Fig. S5b). We speculate that the difference may become more obvious if observed for a longer period. Besides sWAT, the weight of BAT was decreased in mice treated with BK for 14 days (Fig. S5c). No differences were observed in the blood glucose and serum insulin levels between mice treated with or without BK for 14 days (Fig. S5d, e). BK treatment for 5 or 10 days did not affect serum free fatty acids (FFAs) levels (Fig. S5f). However, serum FFAs levels were decreased in mice treated with BK for 14 days (Fig. S5f). In addition, O_2_ consumption was increased in BK-treated mice in the absence of any changes in food intake and locomotor activity (Fig. S5g, h). As was the case after a single BK injection, the body temperature was higher in mice treated with BK than in untreated mice throughout the study (Fig. S5i). UCP1 was also upregulated in BAT of mice after BK injection for 14 days (Fig. S5j).

To confirm the role of UCP1 in BK-mediated effects, we first performed time course experiments to elucidate changes in UCP1 expression and body temperature at room temperature. We measured the body temperature within 30 mins post-injection of BK, and no differences in body temperature were observed between the two groups at 15 min after BK injection (Fig. S6a). BK significantly increased body temperature starting at 20 min after injection (Fig. S6a). Additionally, UCP1 expression was not changed in the BAT at 10 min after injection (Fig. S6b), but increased at 15 min after injection (Fig. S6c). Therefore, changes in UCP1 expression preceded the BK-induced rise in body temperature. Finally, WT and UCP1 null (*Ucp1*^-/-^) mice were treated with a single injection of 1 mg/kg BK. BK-treated WT mice displayed a significantly higher body temperature at room temperature and under acute cold conditions, whereas these effects were attenuated in *Ucp1*^-/-^ mice (Fig. 3h, S6d). These findings suggested that UCP1 plays an important role in the BK-regulated body temperature. Consistently with these results, UCP1 knockout attenuated BK-increased oxygen consumption in primary brown adipocytes (Fig. S6e, S6f).

### Bradykinin upregulates uncoupling protein 1 (UCP1) via the bradykinin receptor B2 (B2R)

BK exerts its action through the bradykinin B2 receptor (B2R). To assess the functions of B2R, we treated mice with the B2R antagonist icatibant^22^ upon acute cold exposure or at room temperature. We found that UCP1 was downregulated in the BAT of icatibant-treated mice in response to acute cold exposure (Fig. S7a). Similar results were observed in the sWAT (Fig. S7b, S7c), suggesting that icatibant protected against the cold-induced browning of sWAT. The mRNA abundance of other genes related to WAT browning was decreased in the sWAT of icatibant-treated mice in response to acute cold exposure (Fig. S7b). Consistently, a distinct histological morphology characterized by enlarged lipid droplets was observed in the sWAT of icatibant-treated mice in response to acute cold exposure (Fig. S7d). Accordingly, icatibant reduced body temperature during acute cold exposure (Fig. S7e). However, icatibant had no effect on these parameters at room temperature (Fig. S7a–e). Some other physiological parameters were also measured in these mice (Fig. S7f–i). There were no differences in body weight or serum insulin levels among the four groups at the end of the experiments (Fig. S7f, 7h). Serum FFAs levels were increased in mice under acute cold exposure but not affected by icatibant (Fig. S7i).

**Fig. 4:**
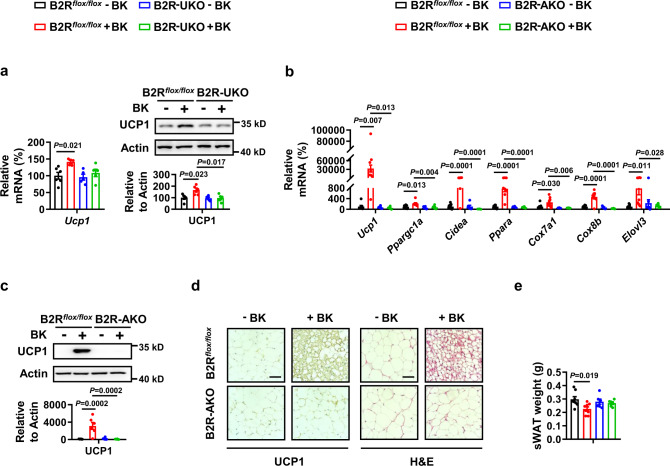
Bradykinin increases uncoupling protein 1 (UCP1) expression via bradykinin receptor B2 (B2R). **a** *Ucp1* mRNA (**a,** **left**) and protein levels (**a,** **right**) in BAT of B2R^*flox/flox*^ or B2R-UKO male mice i.p. injected with a single dose of PBS (–BK) or 1 mg/kg BK ( + BK) for 30 mins in the absence of food and water at 25 °C. **b**–**e** mRNA levels of browning marker genes in sWAT (**b**), UCP1 protein levels in sWAT (**c**), representative immunohistochemistry (IHC) staining of UCP1 in sWAT (**d left**), representative H&E staining of sWAT (**d right**), and sWAT weight (**e**) from B2R^*flox/flox*^ or B2R-AKO male mice i.p. injected with PBS (–BK) or 1 mg/kg BK ( + BK) once a day for 14 days at 25 °C. Scar bars, 50 μm (**d**). For **a**, *n* = 6 (B2R^*flox/flox*^–BK) and 5 (B2R^*flox/flox*^ + BK, B2R-UKO–BK, B2R-UKO + BK) mice per group (left); *n* = 5 in each group (right). For **b**, *n* = 8 (B2R^*flox/flox*^–BK, B2R^*flox/flox*^ + BK) or 6 (B2R-AKO–BK, B2R-AKO + BK) mice per group. For **c**, *n* = 6 in each group. For **e**, *n* = 8 (B2R^*flox/flox*^–BK, B2R^*flox/flox*^ + BK) and 6 (B2R-AKO–BK, B2R-AKO + BK) mice per group. Mean±SEM are representative of at least two independent experiments (**a,** **b,** **c,** **e**); ordinary two-way ANOVA with Tukey’s multiple comparisons test (**a,** **b,** **c,** **e**). Source data are provided as a Source data file.

To further assess the function of B2R in adipose tissues, we generated mice ablating B2R selectively in the adipose tissue by crossing Bdkrb2^flox/flox^ mice with Ucp1-Cre lines to knockout B2R in BAT (B2R-UKO, Fig. S8a–c) or Adipoq-Cre lines to knockout B2R in BAT and WAT (B2R-AKO, Fig. S9a–c), respectively. We measured the body weight, food intake, body temperature, and energy expenditure of B2R-UKO mice at room temperature. None of these parameters differed between control and B2R-UKO mice (Fig. S8d–g). When challenged with a single BK injection at room temperature, B2R-UKO mice displayed lower UCP1 expression in BAT (Fig. 4a). Consistently with this, BK-treated control mice exhibited a significantly higher body temperature, whereas no differences in body temperature were observed between B2R-UKO mice treated with or without a single 1 mg/kg BK injection at room temperature (Fig. S10a).

Further, body weight, food intake, body temperature, and energy expenditure at room temperature were not altered in B2R-AKO mice (Fig. S9d–g). However, B2R-AKO mice exhibited reduced sWAT browning compared to control mice in response to 1 mg/kg BK injection once a day for 14 days at room temperature, as evidenced by hematoxylin and eosin (H&E) staining and the expression of browning markers (Fig.4b–e). No differences were observed in the food intake, body weight, blood glucose levels, and serum insulin levels among the four groups (Fig. S10b–f).

### Adrenergic signaling and nitric oxide (NO) signaling are involved in bradykinin-increased UCP1 expression

**Fig. 5:**
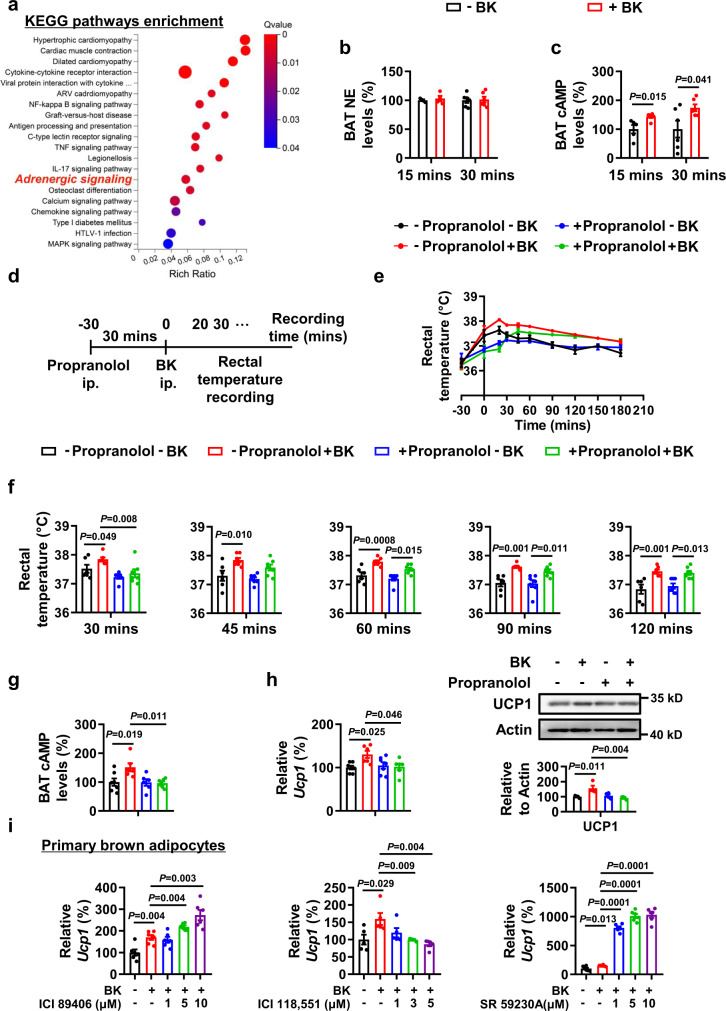
Effects of β-adrenergic blockers on the actions of BK. **a–c** RNA-sequencing analysis (**a**), norepinephrine (NE, **b**) and cAMP (**c**) levels of BAT from male WT mice i.p. injected with a single dose of PBS (–BK) or 1 mg/kg BK (+BK) for 15 or 30 mins in the absence of food and water at 25 °C. **d–h** Mice received PBS (–propranolol) or 5 mg/kg propranolol (+propranolol) 30 mins before the injection of PBS (–BK) or 1 mg/kg BK (+BK) for 15 mins in the absence of food and water at 25 °C. Rectal temperature at different time point (**e,** **f**), cAMP levels (**g**) and UCP1 (**h**) expression of BAT were examined. The rectal temperature values from **e** were shown in **f** to accurately reflect the changes at different time point. **i** *Ucp1* mRNA levels of primary brown adipocytes treated with or without antagonists in the presence of 0.1 μM BK for 24 h. For **a**, *n* = 4 per group. For **b**, *n* = 4 or 6 per group. For **c**, *n* = 5 or 6 per group. For **e**–**g**, *n* = 6 or 7 per group. For **h** *n* = 4-7 per group. For **i**, *n* = 6 (left and right) and *n* = 5 (middle) per group. Data are represented as mean ± SEM; two-tailed unpaired Student’s *t*-test (**b,** **c**), two-way RM ANOVA with Geisser-Greenhouse’s correction followed by Tukey’s multiple comparisons test (**e**), ordinary two-way ANOVA with Tukey’s multiple comparisons test (**f–h**). For **e**, –propranolol–BK vs. –propranolol+BK *p* = 0.0002, –propranolol–BK vs. +propranolol –BK *p* = 0.506, –propranolol+BK vs. +propranolol+BK *p* = 0.002, +propranolol–BK vs. +propranolol+BK *p* = 0.099. Source data are provided as a Source data file.

To delineate the mechanisms underlying the increased UCP1 expression by BK, RNA-sequencing analysis was performed (Fig. 5a, Supplementary Data 1). Most notably, Kyoto Encyclopedia of Genes and Genomes (KEGG) enrichment analysis revealed that the adrenergic signaling pathway was enriched remarkably in BAT of mice injected with BK. Interestingly, a study shows that B2R can physically interact with beta 2 adrenergic receptor (β2AR) and forms a functional heterodimer. As a result, stimulation with BK can rapidly (within 5 mins) transactivate β2AR via B2R^23^. Therefore, we speculated that the rapid effects of BK on UCP1 expression and thermogenesis may be mediated by adrenergic signaling. To explore this possibility, we measured the levels of NE and cAMP, reflecting adrenergic signaling^24^, in the BAT of mice injected with BK for 15 or 30 mins. Though NE levels in the BAT remained unchanged following BK injection (Fig. 5b), cAMP levels were much higher in BK-treated mice at both time points (Fig. 5c), suggesting that adrenergic signaling may be acutely activated by BK independently of NE stimulation. To determine whether adrenergic signaling contributes to the effects of BK, we treated mice with β-adrenoceptor antagonist propranolol^25^, prior to BK injection. Propranolol significantly blocked the acute effects of BK on body temperature with the effects lasted for an hour, and the levels of cAMP and UCP1 expression in BAT (Fig. 5d–h). These results suggest that βAR was involved in the acute effects of BK in thermogenesis.

Three subtypes of βARs are expressed in BAT^26^. To further confirm the role of βARs in this regulation, we examined the effects of antagonists targeting different subtypes of βARs on BK’s effects. Three antagonists include ICI 89406, ICI 118551, SR59230A, which are reported to block β1AR, β2AR or β3AR, respectively^27–29^. BK’s activation of Ucp1 expression was largely blocked by β2AR antagonist ICI 118551, but β1AR and β3AR antagonists had no effect (Fig. 5i). Consistently, it is reported that β2ARs are expressed in human brown adipose tissue and responsible for thermogenesis^28^, though most commonly known subtypes are β1ARs and β3ARs. Furthermore, it is reported that β2AR is transactivated by BK^23^. These results suggest that BK may function via β2ARs.

**Fig. 6:**
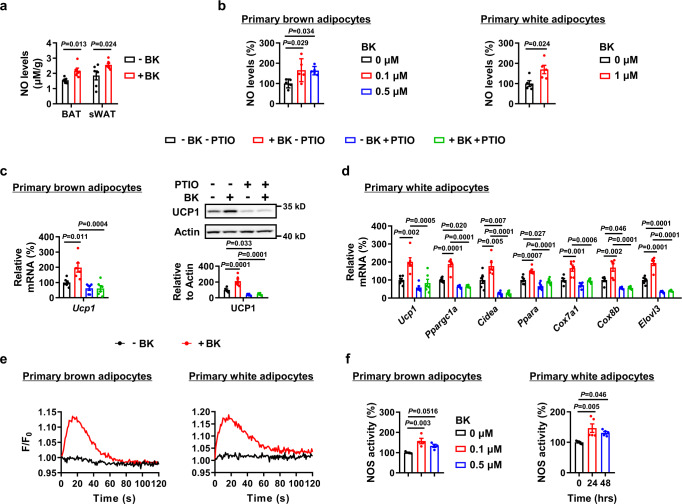
Nitric oxide (NO) signaling is involved in bradykinin-increased UCP1 expression. **a** NO levels of BAT and sWAT from male WT mice i.p. injected with PBS (–BK) or 1 mg/kg BK ( + BK) for 30 mins or 14 days, respectively. **b** NO levels of primary brown adipocytes treated with 0, 0.1 or 0.5 μM BK for 48 h (left). NO levels of primary white adipocytes treated with 0 or 1 μM BK for 48 h (right). **c** *Ucp1* mRNA (left) and protein levels (right) of primary brown adipocytes treated with (+PTIO) or without (–PTIO) 50 μM PTIO in the absence (–BK) or presence (+BK) of 0.1 μM BK for 48 h. **d** mRNA levels of browning marker genes in primary white adipocytes treated with (+PTIO) or without (–PTIO) 100 μM PTIO in the absence (–BK) or presence (+BK) of 1 μM BK for 48 h. **e** Ca^2+^ changes in primary brown (left) or white (right) adipocytes treated with 0.1 μM or 1 μM BK, respectively. **f** Nitric oxide synthase (NOS) activity in primary brown adipocytes treated with 0, 0.1 or 0.5 μM BK for 48 h (left), or in primary white adipocytes treated with 1 μM BK for different amounts of time (right). For **a**, *n* = 5 (–BK BAT), 6 (–BK sWAT, +BK BAT) and 8 (+BK sWAT) mice per group. For **b**, *n* = 5 in each group. For **c** and **d**, *n* = 6 in each group. For **f**, *n* = 4 (left) or 5 (right) in each group. Mean±SEM are representative of at least two independent experiments (**a**) or at least three independent experiments (**b**–**f**); two-tailed unpaired Student’s *t*-test (**a,** **b** right), ordinary one-way ANOVA with Dunnett’s test (**b** left, **f**), ordinary two-way ANOVA with Tukey’s multiple comparisons test (**c,** **d**). Source data are provided as a Source data file.

Based on these results, we speculated that the acute effects of BK are possibly mediated by adrenergic signaling, most likely dependent on the transactivation of β2AR by BK. However, the chronic effects of BK are mediated by other mechanisms, as BK still increased body temperature in the presence of propranolol 60 min after injection. Especially, pathways for BK and nitric oxide (NO) signaling were of particular interest to us, because NO has been shown to upregulate UCP1 in adipose tissues^30^. We found that BK could increase NO levels in both BAT and sWAT (Fig. 6a). Similar results were observed in vitro (Fig. 6b). Sequestration of NO by 2-phenyl-4,4,5,5- tetramethylimidazoline-1-oxyl 3-oxide (PTIO) negated BK-induced UCP1 expression in primary brown adipocytes and the expression of browning markers in primary white adipocytes (Fig. 6c, d), indicating that NO signals are possibly involved in BK-increased UCP1 expression. In addition, B2R knockout in adipose tissue decreased BK-induced NO levels (Fig. S11a, 11b). Ca^2+^-nitric oxide synthase (NOS) is the classical pathway to generate NO. Consistently, we observed elevation of intracellular Ca^2+^ levels and NOS activity in both primary brown and white adipocytes treated with BK (Fig. 6e, f).

### Acute cold exposure increases bradykinin contents by decreasing hepatic prolyl endopeptidase (PREP) activity

**Fig. 7:**
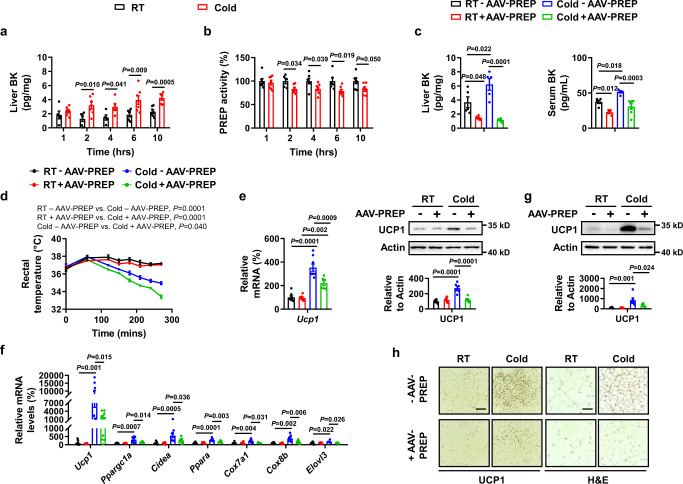
Acute cold exposure decreases hepatic prolyl endopeptidase (PREP) activity and the effects of PREP overexpression. **a** BK levels in the liver of male WT mice exposed to 25 °C (RT) or 4 °C (Cold) for different amounts of time in the absence of food and water. **b** PREP activity in the liver of male WT mice exposed to 25 °C (RT) or 4 °C (Cold) for different amounts of time in the absence of food and water. **c**–**h** Hepatic BK levels (**c** left), serum BK levels (**c** right), rectal temperature (**d**), *Ucp1* mRNA and protein levels in BAT (**e**), mRNA levels of browning marker genes in sWAT (**f**), UCP1 protein levels in sWAT (**g**), representative immunohistochemistry (IHC) staining of UCP1 in sWAT (**h** left), and representative H&E staining of sWAT (**h** right) from WT male mice infected with (+AAV-PREP) or without (–AAV-PREP) AAV-PREP at 25 °C (RT) or 4 °C (Cold) for 2 h in the absence of food and water. Scar bars, 50 μm (**h**). For **a**, *n* = 5 (RT 1 h, RT 4 h, Cold 4 h), 6 (RT 2 h, Cold 1 h, Cold 10 h) and 7 (RT 6 h, RT 10 h, Cold 2 h, Cold 6 h) mice per group. For **b**, *n* = 7 (RT 1 h, RT 4 h, RT 10 h, Cold 1 h, Cold 4 h, Cold 6h), 5 (RT 2 h) and 6 (RT 6 h, Cold 2 h, Cold 10 h) mice per group. For **c**, *n* = 5 (liver RT–AAV-PREP, RT + AAV-PREP, Cold–AAV-PREP; serum RT–AAV-PREP, RT + AAV-PREP, Cold–AAV-PREP), 6 (liver Cold+AAV-PREP) and 7 (serum Cold+AAV-PREP) mice per group. For **d** *n* = 6 (RT–AAV-PREP), 8 (RT + AAV-PREP), 5 (Cold–AAV-PREP) and 7 (Cold+AAV-PREP) mice per group. For **e**, *n* = 8 (RT–AAV-PREP), 7 (RT + AAV-PREP), 8 (Cold–AAV-PREP) and 6 (Cold+AAV-PREP) mice per group (left); *n* = 6 in each group (right). For **f**, *n* = 8 (RT–AAV-PREP, Cold–AAV-PREP), 7 (RT + AAV-PREP) or 5-8 (Cold+AAV-PREP) mice per group. For **g**, *n* = 6 in each group. Mean±SEM are representative of at least two independent experiments (**a–g**); two-tailed unpaired Student’s *t*-test (**a,** **b**), ordinary two-way ANOVA with Tukey’s multiple comparisons test (**c,** **e,** **f,** **g**), two-way RM ANOVA with Geisser-Greenhouse’s correction followed by Tukey’s multiple comparisons test (**d**). Source data are provided as a Source data file.

The levels of BK in serum are determined by a balance between BK formation and degradation^31^. BK is generated through the cleavage of HMWK by plasma kallikrein and is degraded by several enzymes; the liver is the main organ for its degradation^32^. We first measured serum levels of the BK precursor HMWK in mice at room temperature or under conditions of acute cold exposure for different periods of time. However, no changes in serum HMWK levels were observed (Fig. S12a), suggesting that acute cold-increased serum BK levels are unlikely because of increased BK production. Next, we measured BK contents in the liver and kidney of mice owing to the roles of these organs in BK degradation^32^. We found that liver BK levels were elevated in response to acute cold at each time point examined, whereas the BK content was not changed in the kidney (Fig. 7a, S12b). These results indicated that the liver may play a role in increased serum BK levels in response to acute cold, supporting a previous study in which the liver was shown to have a large capacity to degrade BK^32^. Several enzymes, including PREP, carboxypeptidases N (CPN), X-prolyl aminopeptidase (aminopeptidase P) 1 (XPNPEP1), thimet oligopeptidase 1 (THOP1), and membrane metallo endopeptidase (MME)^32,33^ have been reported to cleave BK and express at high levels in the liver, we therefore examined the expression of these enzymes in the liver following acute cold exposure. Among the genes examined, only *Prep* expression was reduced (Fig. S12c). Furthermore, acute cold exposure inhibited PREP activity (Fig. 7b). PREP cleaves peptide bonds on the carboxylic sides of prolyl residue within BK. We then used adeno-associated viruses (AAV) to overexpress mouse PREP for enhancing PREP activity in the liver. Four weeks after the tail vein administration of AAV8 encoding PREP (AAV8-thyroid hormone binding protein [TBG]-PREP), we observed increased expression and activity of PREP (Fig. S12d). AAV8-TBG-PREP lowered hepatic and serum BK levels (Fig. 7c). Moreover, mice injected with AAV8-TBG-PREP were unable to maintain their core body temperature when challenged with an acute cold tolerance test and displayed decreased UCP1 expression in BAT (Fig. 7d, e). Overexpression of hepatic PREP also blocked acute cold-induced sWAT browning, as demonstrated by H&E staining, as well as the expression of browning markers (Fig. 7f–h).

### Oleic acid decreases prolyl endopeptidase (PREP) activity and increases bradykinin levels

**Fig. 8:**
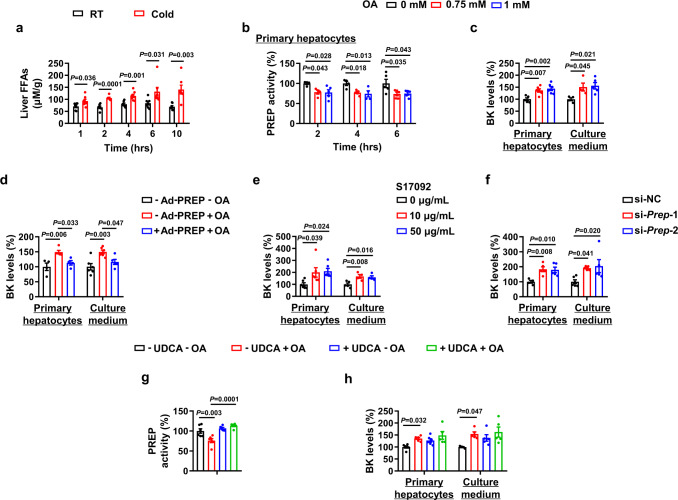
Oleic acid decreases prolyl endopeptidase (PREP) activity and increases bradykinin levels. **a** Free fatty acids (FFAs) levels in the liver of male WT mice exposed to 25 °C (RT) or 4 °C (Cold) for different amounts of time in the absence of food and water. **b** PREP activity in primary hepatocytes treated with 0, 0.75 or 1 mM oleic acid (OA) for different amounts of time. **c** BK levels in cell lysates and the culture medium of primary hepatocytes treated with 0, 0.75 or 1 mM OA for 6 h. **d** BK levels in cell lysates and the culture medium of primary hepatocytes infected with (+ Ad-PREP) or without (–Ad-PREP) Ad-PREP in the absence (–OA) or presence (+OA) of 1 mM OA for 6 h. **e** BK levels in cell lysates and the culture medium of primary hepatocytes treated with 0, 10 or 50 μg/mL S17092 for 6 h. **f** BK levels in cell lysates and the culture medium of primary hepatocytes transfected with (si-*Prep*) or without (si-NC) *Prep* siRNA for 72 h. **g**, **h** PREP activity (**g**) and BK levels in cell lysates and the culture medium of primary hepatocytes (**h**) treated with (+UDCA) or without (–UDCA) ursodeoxycholic acid (UDCA) in the absence (–OA) or presence (+OA) of 1 mM OA for 6 h. For **a**, *n* = 7 in each group. For **b** and **c**, *n* = 4 or 5 in each group. For **d**, *n* = 4–6 in each group. For **e**, *n* = 6 in each group for hepatocytes; *n* = 5 (medium 0 μg/mL S17092) and 4 (medium 10 μg/mL, 50 μg/mL S17092) in each group. For **f**, *n* = 4–6 in each group. For **g**, *n* = 6 in each group. For **h**, *n* = 5 or 6 in each group for hepatocytes; *n* = 5 in each group for medium. Mean±SEM are representative of at least two independent experiments (**a**) or at least three independent experiments (**b–h**); two-tailed unpaired Student’s *t*-test (**a**), ordinary one-way ANOVA with Dunnett’s test (**b,** **c,** **e,** **f**), ordinary one-way ANOVA with Tukey’s test (**d**), ordinary two-way ANOVA with Tukey’s multiple comparisons test (**g,** **h**). Source data are provided as a Source data file.

We then investigated the upstream signals that inhibit PREP activity during acute cold exposure. Cold exposure is known to increase the levels of hepatic FFAs, which affect PREP activity^34^, suggesting a possible effect of FFAs on hepatic PREP activity and BK content during acute cold exposure. During acute cold exposure, hepatic FFAs and triglyceride levels were increased (Fig. 8a, S13a). To test whether FFAs play an important role in inhibiting PREP activity and BK levels, we treated primary hepatocytes with control vehicle or oleic acid, the major components of FFAs found in serum^35^. We found that OA inhibited PREP activity (Fig. 8b) and increased BK content in primary hepatocytes and corresponding medium (Fig. 8c). Furthermore, OA-increased BK content was blocked by overexpression of PREP (Fig. 8d, S13b). PREP inhibition also increased the BK content in primary hepatocytes and corresponding medium (Fig. 8e, f, S13c, S13d). To further confirm the role of OA, primary hepatocytes were treated with ursodeoxycholic acid (UDCA), a potent liver-specific fatty acid transport protein 5 (FATP5) inhibitors^36^, in the presence or absence of OA. As previously reported, UDCA inhibited OA uptake by primary hepatocytes (Fig. S13e, f). UDCA also blocked OA-decreased PREP activity and -increased BK content (Fig. 8g, h).

CL-316,243 is a selective β3 adrenergic receptor agonist which stimulates lipolysis in adipocytes^37^. In mice treated with 1 mg/kg CL-316,243 or vehicle control for 3 or 5 h, CL-316,243 increased liver FFAs content (Fig. S14a). Consistently, hepatic PREP activity decreased, whereas hepatic and serum BK levels increased in mice injected with CL-316,243 (Fig. S14b, c). We also observed decreased hepatic PREP activity in young mice but not in aged mice in response to acute cold exposure (Fig. S3d).

### Angiotensin-converting enzyme inhibitors (ACEIs) induce BAT UCP1 expression and WAT browning via B2R

**Fig. 9:**
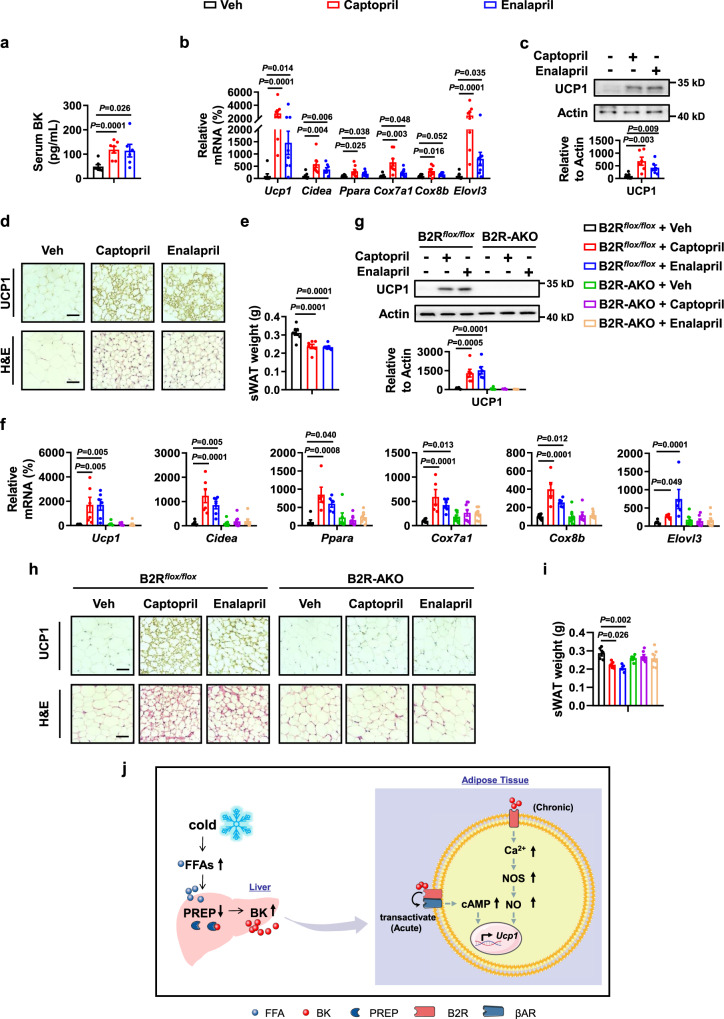
Angiotensin-converting enzyme inhibitors (ACEIs) induce WAT browning via B2R. **a**–**e** Serum BK levels (**a**), mRNA levels of browning marker genes in sWAT (**b**), UCP1 protein levels in sWAT (**c**), representative immunohistochemistry (IHC) staining of UCP1 in sWAT (**d up**), representative H&E staining of sWAT (**d bottom**), and sWAT weight (**e**) from WT male mice treated with 40 mg/kg/day captopril or 70 mg/kg/day enalapril by gavage once a day for 21 days. **f**–**i** mRNA levels of browning marker genes in sWAT (**f**), UCP1 protein levels in sWAT (**g**), representative immunohistochemistry (IHC) staining of UCP1 in sWAT (**h** up), representative H&E staining of sWAT (**h** bottom), and sWAT weight (**i**) from B2R^*flox/flox*^ or B2R-AKO male mice treated with 40 mg/kg/day captopril or 70 mg/kg/day enalapril by gavage once a day for 21 days. Scar bars, 50 μm (**d,** **h**). **j** Working model. For **a**, *n* = 7 (Veh, Captopril) and 6 (Enalapril) mice per group. For **b**, *n* = 8 in each group. For **c**, *n* = 7 (Veh) and 6 (Captopril, Enalapril) mice per group. For **e**, *n* = 8 (Veh) and 7 (Captopril, Enalapril) mice per group. For **f**, *n* = 5-7 (B2R^*flox/flox*^ + Veh), 5 or 6 (B2R^*flox/flox*^ + Captopril, B2R^*flox/flox*^ + Enalapril), 5-8 (B2R-AKO + Veh, B2R-AKO + Captopril, B2R-AKO + Enalapril). For **g**, *n* = 6 in each group. For **i**, *n* = 5 (B2R^*flox/flox*^ + Veh, B2R^*flox/flox*^ + Captopril, B2R^*flox/flox*^ + Enalapril), 6 (B2R-AKO + Veh) and 7 (B2R-AKO + Captopril, B2R-AKO + Enalapril) mice per group. Mean±SEM are representative of at least two independent experiments (**a–c,** **e,** **f,** **g,** **i**); two-tailed unpaired Student’s *t*-test (**a–c,** **e**), ordinary two-way ANOVA with Tukey’s multiple comparisons test (**f,** **g,** **i**). Source data are provided as a Source data file.

Based on the above results, we speculated that manipulating BK content pharmacologically via reduction of BK degradation might have effects similar to those of BK treatment. To test this possibility, we treated mice with ACEIs, a group of medicines mainly used to treat certain heart and kidney conditions, or control vehicle, and examined the effect of ACEIs on UCP1 expression in BAT and WAT. BK can be metabolized by ACE and ACEIs could increase BK levels via blocking its breakdown. Mice were treated with two ACEIs, captopril (40 mg/kg) and enalapril (70 mg/kg)^38,39^, by gavage once a day for 21 days at room temperature. As expected, both captopril and enalapril increased serum BK levels (Fig. 9a, S15a) in the absence of changes in food intake (Fig. S15b). Both captopril and enalapril increased BAT UCP1 expression and body temperature (Fig. S15c, d). Moreover, captopril and enalapril induced sWAT browning and decreased the weight of sWAT, as demonstrated by the corresponding changes in the expression of WAT browning markers, as well as H&E staining (Fig. 9b–e). These ACEIs-induced changes in BAT UCP1 expression and sWAT browning were blocked in B2R-AKO mice (Fig. 9f–i, S15e).

## Discussion

Here, we first measured the serum kinins levels during acute cold exposure. We found that the levels of both BK and its metabolite, des-Arg^9^-BK, increased in response to 2 h of acute cold exposure. However, the levels of serum KLP and its metabolite, des-Arg^10^-KLP, did not change at this time point. These data suggest that BK may be the first kinin to respond to acute cold exposure. Similarly, circulating concentrations of BK have been shown to increase significantly as body temperature decreases in humans^40^. Our further study focused on BK and demonstrated a role for BK in regulating body temperature during acute cold exposure, as treatment with anti-BK antibodies reduced body temperature while BK had the opposite effect. However, we found that the results differed in response to chronic cold exposure. Indeed, serum BK levels were lower in mice exposed to 4 °C for 1 week than in those maintained at room temperature (Fig. S16a). The changes in circulating BK levels differed between acute and chronic cold exposure, possibly because chronic cold exposure is a more complex stressor. For acute cold exposure experiments, mice were caged individually in the absence of food, water or bedding. For chronic cold exposure experiments, mice were caged individually in the presence of food, water and bedding. Consistent with previous reports^41^, we found that chronic cold exposure increased food intake markedly (Fig. S16b). Therefore, the effects of chronic cold exposure on circulating BK levels are determined by several factors, including cold and food intake.

Cold exposure activates brown and beige adipocytes to dissipate heat for NST to maintain body temperature. The function of BK in thermogenesis is poorly understood. Here, we provide evidence showing that BK significantly activated BAT activity and induced WAT browning, as demonstrated by the changes in infrared images, OCR, and other parameters examined. Similar effects have been observed in vitro, suggesting a direct effect of BK in thermogenesis. UCP1 mediates NST and is induced in BAT and WAT in response to cold exposure. We found that BK increased UCP1 expression in vivo and in vitro. We assume that the response of cells in vitro to BK is much slower than BAT in vivo, because the differentiated primary brown adipocytes are not exactly the same as those in BAT. Preadipocytes are isolated from BAT and differentiated into adipocytes displaying brown adipocytes features when exposed to media supplemented with some stimulus. The approach may lead to changes in adipocyte characteristics compared with in vivo^42^. In addition, the microenvironments in vivo and in vitro are quite different. Many other cell types and nerves exist within BAT that may contribute to the phenotypes observed in mice treated with BK. Therefore, different time periods may be needed for BK to exert its function in vivo and in vitro. We also provided evidence showing that changes in BAT UCP1 expression preceded the BK-induced rise in body temperature, and the effects of BK were attenuated in UCP1 null mice. Similar phenomena have been observed in other studies. For example, CL316,243 could increase body temperature 10 mins after injection, and this rise was blunted in UCP1 null mice^43–45^. Norepinephrine has been shown to induce thermogenesis within 20 mins in WT mice, and the response to norepinephrine in UCP1-ablated mice was much weaker than that in WT mice^46,47^. However, the rapid increase in the body temperature following BK injection may not be driven solely by the induction of UCP1 expression, as the molecular differences in brown fat mitochondria between WT and UCP1 null mice extend substantially beyond the deletion of UCP1 itself^48^. In fact, the results from our BAT RNA-Sequencing analysis of mice injected with BK also indicate other underlying mechanisms. Further studies are needed to investigate this issue. Both our study and previous reports have clarified the role of KKS in thermogenesis; however, the effects of individual KKS components on thermogenesis may be different^49^. In contrast to BK, kininogens repress BAT activity in rats^49^, suggesting the complexity of the system in this regard.

Two distinct kinin receptor subtypes, B1 and B2, have been identified; both belong to the rhodopsin family of G protein-coupled receptors. The B2R is widely and constitutively expressed in central and peripheral tissues and mediates the action of BK and kallidin. Their metabolites, des-Arg^9^-BK and des-Arg^10^-kallidin, are the preferential agonists of B1R. B1R is expressed at a very low level in healthy condition but induced after injury by various proinflammatory cytokines, such as interleukin-1 β^14^. In the present study, we generated mice specific knockout B2R in adipose tissue using Ucp1-Cre lines or Adipoq-Cre lines, respectively. BK-increased UCP1 expression and body temperature were blocked in these mice. Similarly, B2R antagonist icatibant decreased UCP1 expression and body temperature during acute cold exposure. Previously, some researchers have attempted to elucidate the role of kinin receptors in adipose tissues using null mice. For example, B1R knockout mice (B1R^-/-^) have less fat content^50^, but rescuing B1R expression exclusively in adipose tissue of B1R^-/-^ mice does not restore the reduced fat mass under normal chow diet (NCD)^51^. B2R null mice (B2R^-/-^) exhibit normal fat mass under NCD and decreased fat content under high fat diet^52^. B1/B2 receptor null-mice display over-activation of BAT and repressed WAT browning^49^. However, the tissue-specific functions of B2R remain elusive. In this study, we found that both B2R-UKO and B2R-AKO mice showed no phenotypic changes in body weight, food intake, body temperature, and UCP1 expression under normal conditions. We assume several possible reasons may account for unchanged UCP1 expression in these mice under basal conditions. First, both BK and kallidin act via B2R. The effects of kallidin on UCP1 expression remain unknown, which needs further investigation. Second, the body does not need to produce much heat in basal condition, and the basal UCP1 expression is rather low. Similar phenomena have been observed in other studies. For example, catecholamines are viewed as major stimulants of cold-induced thermogenesis. However, the UCP1 protein levels in brown adipose tissue of β1/2/3-adrenoceptor knockout mice were not changed at 24  °C compared with wild-type mice^53^. Our observations and others’ work have suggested that each kinin receptor has distinct functions in different tissues.

HMWK is encoded by the gene KNG and produced mainly in the liver. After production, HMWK is released into the blood and cleaved by plasma kallikrein, leading to the generation of BK^31,32^. We found that acute cold exposure didn’t change serum HMWK protein levels. On the other hand, BK is degraded in serum or tissues and can be internalized into and released from cells^54^. It has been shown that the liver has a large capacity to degrade BK^32^. Here we found that liver BK contents were elevated in response to acute cold exposure. When exploring mechanisms underlying cold-increased hepatic BK contents, we focused on FFAs and PREP. It is well-known that cold triggers lipolysis in adipocytes to mobilize lipid stores as FFAs^3^. This leads to increased serum levels of FFAs and causes liver FFAs accumulation, because of the increased hepatic fatty acid uptake during cold exposure^55^. PREP is a cytosolic enzyme highly expressed in the liver and degrades BK at the Pro-Phe bond. FFAs could affect PREP activity directly^34^. Here, we found that cold exposure inhibited PREP activity. PREP over-expression blocked cold-induced changes in hepatic and serum BK levels, BAT activity, WAT browning and body temperature. PREP inhibition increased intracellular and corresponding medium BK levels in vitro. Our findings expand the physiological role of PREP in metabolism and demonstrate that the liver affects circulating levels of BK in response to acute cold exposure. However, we can’t neglect the possibility that other enzymes could also contribute to increased hepatic and serum levels of BK upon acute cold exposure, which needs further investigations. In addition, although BK can increase BAT thermogenesis and body temperature, we found that aged mice exhibited higher serum BK levels than young mice at room temperature. The higher serum BK levels in aged mice may be related to decreased hepatic PREP activity. Alternatively, these observations may be explained by the compensation effect. Because aged mice are hypothermic, they require more BK to sustain their body temperature. Similar phenomena have been reported in other studies. For example, acylcarnitines increase brown fat thermogenesis and body temperature, and aged mice have higher basal levels of acylcarnitines than young mice at room temperature^4^.

Moreover, the effects of FFAs on PREP activity and BK levels were explored in vitro and in vivo. First, treatment with OA reduced PREP activity and increased BK content in primary hepatocytes and corresponding medium. Second, pharmacological inhibition of OA uptake blocked OA-reduced PREP activity and –increased BK contents in cells and medium. Finally, CL-316,243 treatment in mice increased hepatic FFAs contents, raised hepatic and serum BK levels and decreased hepatic PREP activity. CL-316,243 is a β3 adrenergic receptor agonist which stimulates thermogenesis in BAT and lipolysis in WAT^37^. These findings fit with our model upon acute cold challenge. It is now clearly established that brain is a major regulator for thermogenesis upon cold exposure. Recently, researchers found that interaction between other peripheral tissues and adipose tissues also have a key role in thermogenesis in response to cold exposure. The liver is a vital organ that plays a critical role in whole-body physiology via various mechanisms, including secreted factors and neural signals. Our work reveals a cross-talk between the liver and adipose tissue via BK, enabling modulation of body temperature during cold exposure.

As cold exposure promotes BAT activity and WAT browning, both of which could increase whole-body energy expenditure and lead to reduction in fat mass, it is considered to be a method that has beneficial effects on whole-body metabolism. Therefore, there has been great interest in identifying novel circulating factors in response to cold as potential targets for treating obesity and related metabolic diseases. Notably, plasma BK levels are lower in adolescents with obesity^56^. Our data demonstrates that BK could stimulate BAT activity and WAT browning. Additionally, BK has been shown to augment insulin-stimulated glucose uptake of adipocytes in rats and humans^57,58^. Thus, BK may be a potential target for obesity and related metabolic diseases. As BK has a short half-life, we considered the use of drugs for clinical application and focused on ACEIs. ACEIs is a class of drugs for the treatment and prevention of hypertension, heart failure, and other diseases^59^. ACE regulates blood pressure via the renin-angiotensin system, in which renin converts angiotensinogen to angiotensin I (ang I) that is in turn cleaved by ACE to ang II, a potent vasoconstrictor^60^. ACE also breaks down BK, and ACEIs would allow for the continuation of BK^61^. Some reports have indicated a role for ACEIs in reducing fat mass^38,39^, but the mechanisms and effects of ACEIs on BAT activity and WAT browning remain unclear. Here, we found that the two ACEIs, captopril and enalapril, increased the BK levels, promoted BAT activity, and enhanced WAT browning, leading to reduced WAT weight. The effects of ACEIs on BAT activity and WAT browning were blocked in adipose tissue-specific B2R knockout mice, suggesting that BK mediates the effects of ACEIs in reducing fat mass. Our observations provide important insights into the understanding of the ACEIs action.

In summary, our work identifies an important function of BK in regulating BAT thermogenesis and WAT browning during acute cold exposure. Our findings also expand the physiological role of PREP in metabolism, and demonstrate that liver contributes to thermogenesis in response to cold through the degradation of BK (Fig. 9j).

## Methods

### Mice

All mice experiments were performed in accordance with the guidelines of, and under approval by, the Institutional Animal Care and Use Committee at Fudan University (ethical committee approval no.2022030006 S) and the Shanghai Institute of Nutrition and Health, Chinese Academy of Sciences (ethical committee approval nos.SINH-2020-GFF-1, SINH-2021-GFF-1 and SINH-2022-GFF-1). Eight- to ten-week-old male C57BL/6 J mice were used for all experiments except for aged male mice (18-month-old). Wild type (WT), Bdkrb2^*flox/flox*^, Adipoq-Cre, and aged mice were purchased from Shanghai Model Organisms. The UCP1-KO mice were a kind gift from Prof. Xinran Ma (East China Normal University) and the Ucp1-Cre mice were a kind gift from Prof. Jiqiu Wang (Ruijin Hospital, Shanghai Jiaotong University School of Medicine). Mice were housed on a 12 h light/dark cycle from 7 A.M. to 7 P.M. at room temperature (25 °C) and provided free access to standard chow rodent diets (Shanghai Pu Lu Teng Biotechnology, P1103F) and water. Mice were sacrificed by CO_2_ inhalation. For acute cold exposure experiments, mice were caged individually and exposed at 4 °C in the absence of food, water or bedding, while control mice received the same treatment at 25 °C. For mice exposed to cold for 1 week, mice were caged individually in the presence of food, water and bedding. Mice were intraperitoneally (i.p.) injected with a single dose of 10 μg anti-BK or IgG antibodies (Bioss, China) per mouse prior to the experiment. For BK and icatibant treatment, mice were i.p. injected with BK (1 mg/kg body weight; Abcam, UK) or icatibant (1 mg/kg body weight; MCE, China) diluted in PBS. Mice were given an i.p. injection of propranolol (MCE, China) at 5 mg/kg. Administration of CL-316,243 (1 mg/kg body weight; Sigma, USA) or vehicle control of normal saline was performed by i.p. injection^4^. For captopril and enalapril treatment, mice were administrated with 40 mg/kg/day of captopril (MCE, China) or 70 mg/kg/day of enalapril (MCE, China) diluted in ddH_2_O by intragastric injection once a day for 21 days.

### Generation and administration of viruses

To over-express PREP in the liver, adeno-associated viruses (AAV) were purchased from Vigene Biosciences and administered via tail vein injection. WT mice were injected with AAV containing the PREP coding sequence and GFP protein (AAV8-TBG-PREP-GFP), or control viruses only containing GFP protein (AAV8-TBG-GFP) using 1.5625 × 10^12^ vector genomes (vg)/mouse. All experiments were started at 4 weeks after injection. The adenoviruses expressing mouse PREP (Ad-PREP) were purchased from Hanbio Co.Ltd. Cells were infected with Ad-PREP or control adenoviruses at a dose of 10^7^ plaque-forming units/well in 12-well plates for 48 h.

### Rectal temperature and infrared image

Rectal core temperature was measured by the digital thermometer (Physitemp Instruments, USA). BAT temperature was measured using an infrared camera (Magnity Electronics Co., Ltd, China)^9^.

### Primary cell isolation and treatments

Mouse primary hepatocytes were isolated using Type I collagenase (Worthington, USA) perfusion from mice aged 8 weeks^62^. OA (Sigma, USA), s17092 (Sigma, USA) and UDCA (MCE, China) were added at the indicated concentrations in DMEM without FBS on the second day following hepatocytes isolation. Double-stranded siRNA targeting mouse PREP was purchased from GenePharma (Shanghai, China). The siRNA sequence was 5’-GAGCAATGTCCAATCAGAGGTTTAT-3’ and 5’-CGTGTTATATGTGCAAGACTCCTTA-3’. Primary hepatocytes were transfected with PREP siRNA using Lipofectamine 3000 reagent (Invitrogen, USA). To cultivate the primary white and brown adipocytes in vitro, 4-5-week-old or 7-14-day-old mice were utilized for dissecting sWAT and BAT, respectively^63,64^. For white adipocyte differentiation, cells were cultured in the DMEM induction culture medium containing 10 % FBS, 0.5 mM isobutylmethylxanthine (IBMX), 1 µM dexamethasone (Dex), and 1.7 µM insulin for 48 h. After 48 h, cells were cultured in DMEM maintenance medium containing 10% FBS and 1.7 µM insulin until harvest. 1 µM BK (Abcam, UK) was added to the fully differentiated cells for 48 h. For nitric oxide (NO) clearance experiments, 1 µM BK and 100 µM PTIO (Beyotime, China) were added simultaneously for 48 h on day 6. For brown adipocyte differentiation, cells were cultured in the DMEM induction culture medium containing 10% FBS, 0.5 mM IBMX, 5 µM Dex, 1 nM T3, 125 µM indomethacin, and 20 nM insulin for 48h. After 48h, cells were cultured in DMEM maintenance medium containing 10 % FBS, 20 nM insulin, and 1 nM T3 until harvest. 100 nM or 500 nM BK was added to the fully differentiated cells on day 6 for 48 h. ICI 89406 (Topscience, China), ICI 118551 and SR59230A (MCE, China) were used as indicated. For NO clearance experiments, 100 nM BK and 50 µM PTIO were added simultaneously for 48h on day 6. BK was added after the cells were fully differentiated, not during the differentiation. In experiments involving treating cells with BK for 48 h, the medium was refreshed with medium containing BK every 24 h.

### Measurements of plasma, tissue, and cell parameters

Hepatic and cellular lipids were extracted with chloroform/methanol^62,65^. For measurements of hepatic lipids, 20 mg of liver tissue was homogenized in 0.5 mL PBS. After sufficient mixing of 0.4 mL homogenates with 1.6 mL of chloroform/methanol (2:1, v/v), the suspension was centrifuged at 950 x *g* for 10 mins. The lower organic phase was transferred and air-dried overnight in a chemical hood. The residual liquid was re-suspended in 100 μL of 1% Triton X-100 in absolute ethanol, and concentration of TGs or FFAs was determined using a TG kit (SSUF, China) or an FFA kit (Fujifilm, Japan). 5 μL homogenates in PBS was used to determine the protein concentration by BCA Protein Assay Kit (Thermo, USA) for normalization. Membrane proteins were extracted using a kit from Beyotime, China.

If not specified, BK (TSZ Biosciences, USA), des-Arg^9^-BK (TSZ Biosciences, USA), KLP (TSZ Biosciences, USA), des-Arg^10^-KLP (TSZ Biosciences, USA), NE (Nanjing Jiancheng Bioengineering Institute, China), cAMP (TSZ Biosciences, USA), NO content (Beyotime, China) and NOS activity (Beyotime, China) were assessed using kits according to the manufacturers’ protocols. The cross-reactivity of BK ELISA kit for  des-Arg^9^-BK and KLP is negligible.

For experiments in Fig. S1a, Fig. S2 and Fig. S15a, serum or medium was collected in the presence of following inhibitors: 1 mg/mL hexadimethrine bromide (MCE, China), 21 μM aprotinin (MCE, China), 4.5 mM 1,10-phenanthroline monohydrate (MCE, China), 73 μg/mL chicken egg albumin trypsin inhibitor (Merck, USA), 130 nM enalaprilat dihydrate (MCE, China), and 1.6 mg/mL EDTA (Thermo, USA)^66–68^. Samples were collected into low protein binding tubes (Eppendorf, German) with the above inhibitors. BK levels were assessed using LC-MS/MS according to a Waters Corporation case report. 150 μL serum or 1200 μL culture medium spiked with 80 μL 100 ng/mL internal standard (IS) Lys-Des-Arg^9^-BK (Topscience, China) was diluted 1:1 (v/v) in 5% NH_3_·H_2_O. An Oasis WCX 1 cc Vac Cartridge (Waters, USA) was conditioned with 1 mL methanol and then equilibrated with 1 mL 5% NH_3_·H_2_O. The diluted samples were then loaded. Washing was performed using 1 mL 5% NH_3_·H_2_O, followed by 1 mL 10% acetonitrile (ACN) in water (v/v). Elution was conducted twice with 500 μL 1% formic acid in 75:25 ACN:water (v/v/v). The eluant was evaporated and reconstituted in 80 μL of 10:10:80 formic acid/ACN/water (v/v/v). The samples were analyzed using an API 4000 Q-TRAP mass spectrometer (Thermo, USA) coupled to Agilent 1200 HPLC system (Agilent Technologies, USA) with a 5 μm, 4.6 × 150 mm Agilent Zorbox Eclipse XDB-C18 column (Agilent Technologies, USA).

### Mitochondrial function and respiration

Real-time measurements of oxygen consumption rate (OCR) were determined using XF24 Extracellular Flux Analyzer (Seahorse Bioscience, USA). Cells were seeded in the Seahorse XF24 V7 PS Microplates (Agilent Technologies, USA) and then differentiated into white and brown adipocytes separately. For OCR determination, cells were treated with 4 μM oligomycin (Selleck, USA), 2 μM carbonyl cyanide 4-(trifluoromethoxy) phenylhydrazone (FCCP, Sigma, USA), 1 μM antimycin A (ENZO, USA), and 1 μM rotenone (Sigma, USA). Total protein content in each well was quantified using a BCA Protein Assay Kit (Thermo, USA) for normalization. The OCR values were normalized to non-mito OCR and analyzed by Wave Desktop (Agilent Technologies, USA). For measuring the OCR of tissues, freshly isolated tissues were used, and the results were normalized to tissue weight (mg). Tissues (~3 mg) were treated with 30 μM oligomycin, 10 μM FCCP, 15 μM antimycin A, and 5 μM rotenone^63^.

### Measurement of [Ca^2+^]_i_

For determination of the intracellular free Ca^2+^ ([Ca^2+^]_i_), preadipocytes were seeded at approximately 5 × 10^3^ cells/well in black-wall clear-bottom cell plates (Corning, USA), and differentiation was induced. Before measurement, the cell plate was loaded with 5 μM Fluo-4 AM (Beyotime, China) and 2.5 mM probenecid (Aladdin, China) diluted in HBSS (Thermo, USA) for 1 h at 37 °C in the dark. During incubation, 100 nM BK for primary brown adipocytes and 1 μM BK for primary white adipocytes was prepared in compound plate (Abgene, USA). Finally, 10 μL compound was added to each well, and the fluorescence signal was measured for 180 seconds using FLIPR Tetra (Molecular Devices, USA).

### Metabolic parameter measurements

The body composition of mice was determined using magnetic resonance imaging (EchoMRI, USA). Indirect calorimetry was measured by metabolic cage (CLAMS-16; Columbus Instruments, USA).

### Prolyl endopeptidase (PREP) enzyme activity assay

For determination of primary hepatocytes PREP activity, cells were harvested and washed by the assay buffer (10 mM Tris, pH 7.4). Then, the cells were re-suspended in 50 μL assay buffer and homogenized by grinding beads (Servicebio, China) using a homogenizer. After centrifugation, 40 μL supernatant was collected and added to 37.5 μL assay buffer. The samples were then incubated for 30 min at 37 °C. Subsequently, 2.5 μL of 0.6 mM fluorogenic substrate Z-Gly-Pro-AMC (AAT Bioquest, USA) was added and incubated for 40 mins at 37 °C. Reactions were terminated by addition of 50 μL 1 M NaAc (pH 4.2). For determination of mouse hepatic PREP activity, 30 mg liver tissue was homogenized in 500 μL assay buffer. After centrifugation, 10 μL supernatant was collected, added to 465 μL assay buffer, and then incubated for 30 mins at 30 °C. Subsequently, 25 μL of 0.6 mM fluorogenic substrate Z-Gly-Pro-AMC was added and incubated for 20 mins at 30 °C. Reactions were terminated by addition of 500 μL 1 M NaAc (pH 4.2). The standard curve was generated with AMC (AAT Bioquest, USA). Fluorescence was measured using a microplate reader (PerkinElmer, USA) with excitation and emission wavelengths set at 360 and 460 nm, respectively. PREP enzyme activity was normalized by the protein contents^69^.

### Histological analysis

sWAT, epididymal WAT (eWAT), BAT, and liver samples were fixed in 4% paraformaldehyde and stained with hematoxylin and eosin (H&E) or following antibodies: anti-UCP1 (1:500, Abcam, ab209483), BDKRB2 (1:200, Abclonal, A2844), donkey anti-Rabbit IgG Alexa Fluor™ 488 (1:1000, A-21206, Thermo Fisher) antibodies. Nuclei were counterstained with VECTASHIELD® Mounting Medium with 4’,6-diamidino-2 -phenylindole (DAPI, Vector, USA). Images were acquired by Zeiss LSM880 laser scanning confocal microscope or PerkinElmer Vectra platform (PerkinElmer, USA).

### RNA extraction, real-time quantitative PCR (RT-qPCR) and RNA-seq analysis

RNA was extracted from cells and mouse tissues using TRIzol reagent (Invitrogen, USA). Quantitative real-time PCR (RT-qPCR) was determined by ABI QuantStudio™ 6 Flex Real-Time PCR System (Applied Biosystems, USA). The sequences of primers used are listed in Supplementary Table 1. RNA-seq analysis was performed following standard procedures in BGI Genomics, China.

### Immunoblotting and antibodies

Protein was extracted by the RIPA buffer and subjected to the regular western procedure^9^. The following antibodies were used for western blotting: anti-UCP1 (1:1000 for WAT, 1:10000 for BAT, Abcam, ab209483), anti-BDKRB2 (1:1000, ABclonal, A2844), anti-PREP (1:1000, A9838, ABclonal), anti-HMWK (1:500, sc-23914, Santa Cruz), anti-β-Actin (1:3000, 66009-1-Ig, Proteintech), anti-N-Cadherin (1:1000; A19083; ABclonal).

### Data analysis and statistics

Data were represented as “mean±S.E.M.” and analyzed using GraphPad Prism version 8.0 (GraphPad Software, Inc., USA). Two-tailed unpaired Student’s *t*-test was used to compare observations from two groups. For multiple group comparisons, ordinary one-way ANOVA, two-way ANOVA or two-way RM ANOVA with Geisser-Greenhouse’s correction was used, followed by Dunnett’s or Tukey’s multiple comparisons test. Results were considered statistically significant when the *P* value was less than 0.05.

### Reporting summary

Further information on research design is available in the Nature Portfolio Reporting Summary linked to this article.

## Supplementary information

Supplementary Information

Description of Additional Supplementary Files

Supplementary Data 1

Reporting Summary

## Data Availability

The authors declare that all data supporting the findings of this study are available within this paper and its Supplementary Files. The Source data are provided with this paper. The RNA-Seq data have been deposited into the Sequence Read Archive (SRA) in National Center for Biotechnology Information (NCBI) under the accession NO. PRJNA948923. Source data are provided with this paper.

## Source data

Source Data
