# Supplementary material for: Reduced hepatic bradykinin degradation accounts for cold-induced BAT thermogenesis and WAT browning in male mice
Source: Nat Commun. 2023 May 2;14:2523. doi: 10.1038/s41467-023-38141-0 (PMC10154316; doi:10.1038/s41467-023-38141-0)
Supplement: Supplementary file 1 — Supplementary Information [file 41467_2023_38141_MOESM1_ESM.pdf]

Supplementary information for

Reduced hepatic bradykinin degradation accounts for cold-induced BAT thermogenesis and WAT browning in male mice

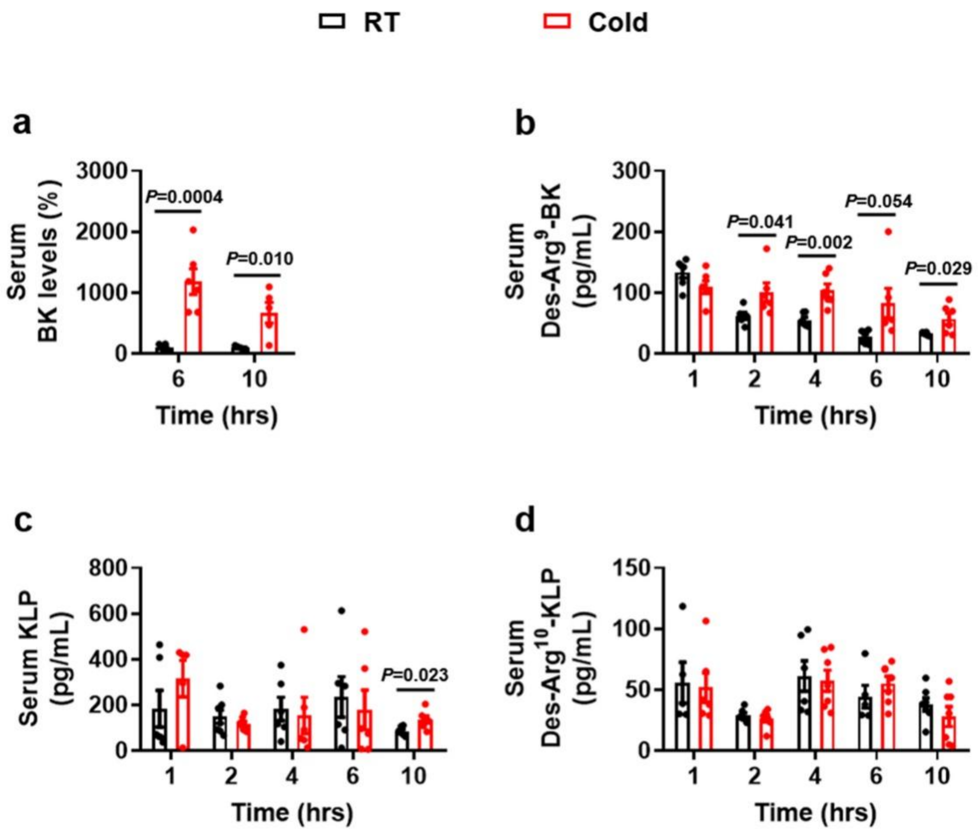

Supplementary Fig. 1 Serum levels of kinins during acute cold exposure

Serum levels of BK (**a**), des-Arg^{9}-BK (**b**), kallidin-like peptide (KLP, Arg-BK) (**c**) and des-Arg^{10}-KLP (**d**) from male WT mice exposed to 25 °C (RT) or 4 °C (Cold) for different amounts of time in the absence of food and water. BK levels were measured by LC-MS/MS, and other kinins were measured by an ELISA kit. For **a,** n = 6 (RT 6hrs, Cold 6hrs) and 5 (RT 10hrs, Cold 10hrs) mice per group. For **b,** n = 6 in each group. For **c**, n = 6 (RT 1hr, 4hrs, 6hrs, 10hrs; Cold 4hrs, 6hrs, 10hrs), 5 (Cold 1hr) and 7 (RT 2hrs; Cold 2hrs) mice per group. For **d**, n = 5 (RT 1hr, 6hrs), 6 (RT 2hrs,

4hrs; Cold 1hr) and 7 (RT 10hrs; Cold 2hrs, 4hrs, 6hrs, 10hrs) mice per group. Mean ± SEM are representative of at least two independent experiments (**a-d**); two-tailed unpaired Student's t-test (**a-d**). Source data are provided as a Source data file.

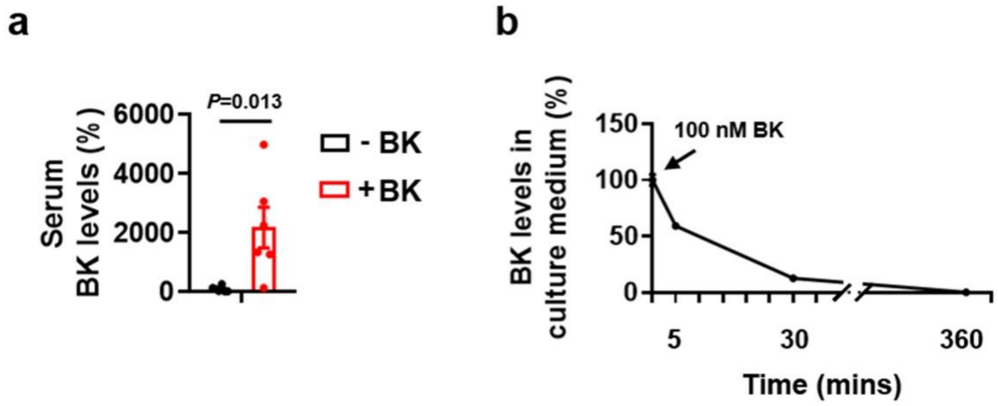

Supplementary Fig. 2 BK levels in the serum of mice injected with BK and culture medium

a Serum BK levels of male WT mice 5 mins after i.p. injection with a single dose of PBS (–BK) or 1 mg/kg BK (+BK) at 25 °C in the absence of food and water. **b** BK levels in primary brown adipocytes culture medium containing 100 nM BK. The culture medium was sampled after 5, 30 or 360 mins. For **a**, n = 6 in each group. For **b**, n = 3 in each group. BK levels were determined by LC-MS/MS. Mean ± SEM are representative of at least two independent experiments (**a**, **b**); two-tailed unpaired Student's t-test (**a**).

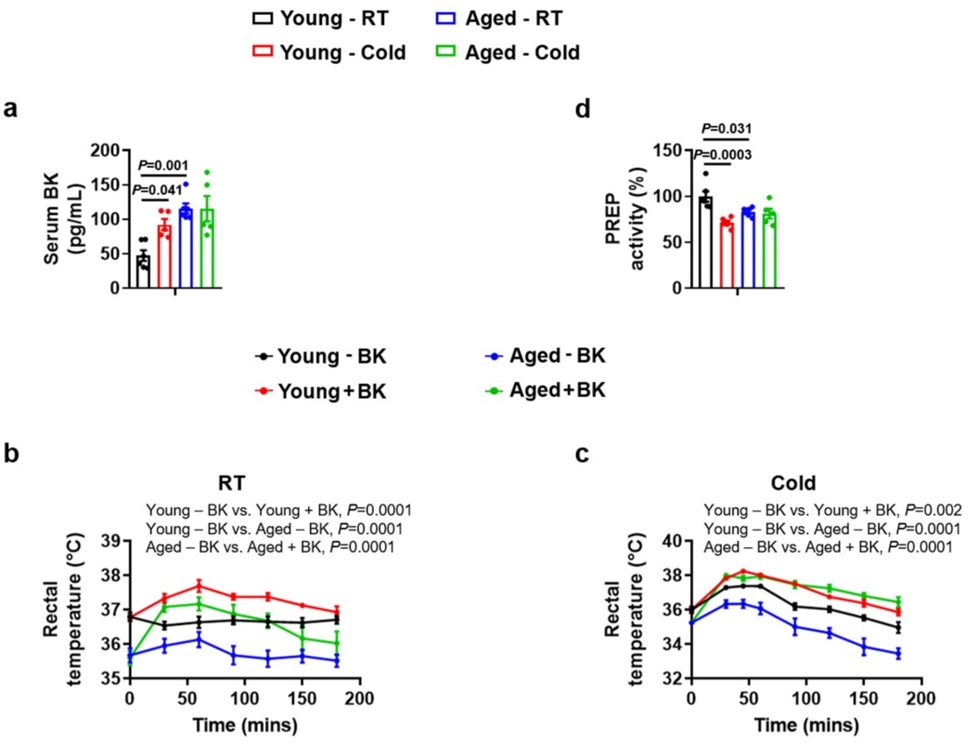

Supplementary Fig. 3 BK treatment protects against age-induced cold sensitivity

a, **d** Serum BK levels (**a**) and hepatic PREP activity (**d**) of male young (10-week-old) and aged (18-month-old) WT mice exposed to 25 °C (RT) or 4 °C (Cold) for 6 hrs in the absence of food and water. **b**, **c** Rectal temperature of male young (10-week-old) and aged (18-month-old) WT mice i.p. injected with a single dose of PBS (–BK) or 1 mg/kg BK (+BK) and exposed to 25 °C (RT, **b**) or 4 °C (Cold, **c**) for different amounts of time. The mice were maintained at RT or exposed to cold immediately after injection in the absence of food and water. For **a**, n = 6 in each group under RT; n = 5 in each group under Cold. For **b**, n = 7 (Young–BK, Aged+BK), 6 (Young+BK) and 5 (Aged–BK) mice per group. For **c**, n = 5 (Young–BK, Aged–BK, Aged+BK) and 7 (Young+BK) mice per group. For **d**, n = 6 (Young–RT, Young–Cold, Aged–RT) and 5 (Aged–Cold) mice per group. Mean ± SEM are representative of at least two independent experiments (**a-d**); ordinary two-way ANOVA with Tukey's multiple

comparisons test (**a**, **d**), two-way RM ANOVA with Geisser-Greenhouse's correction followed by Tukey's multiple comparisons test (**b**, **c**). Source data are provided as a Source data file.

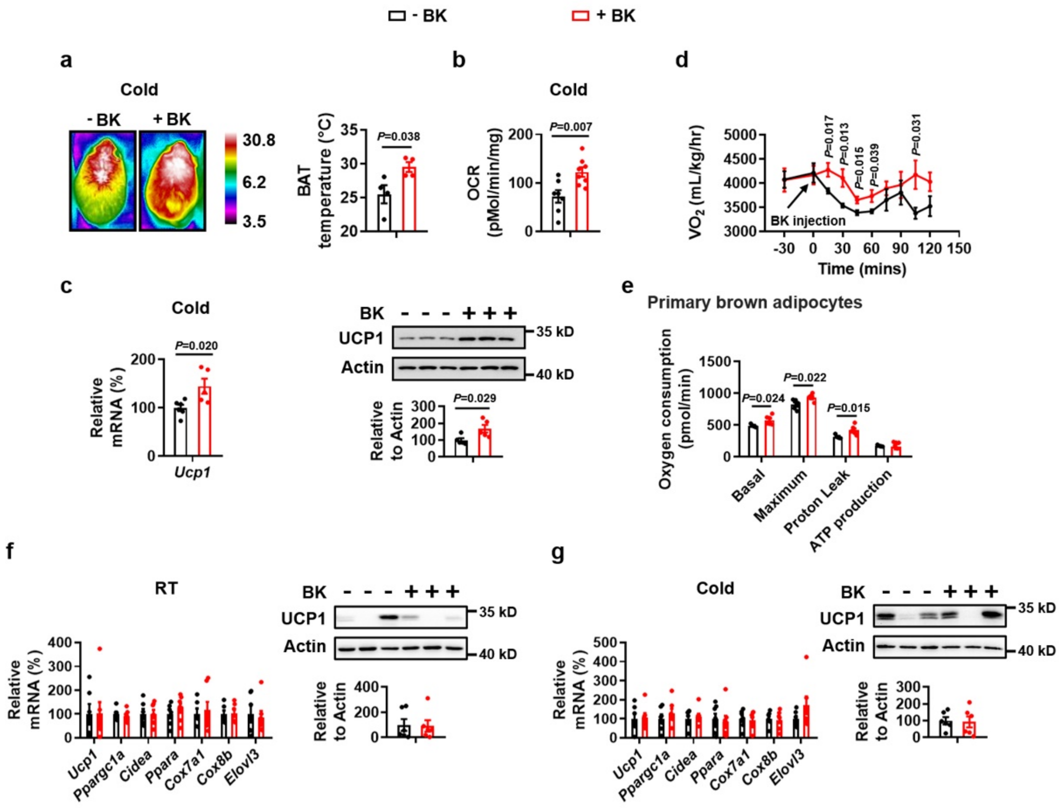

Supplementary Fig. 4 Effects of one BK injection on BAT thermogenesis and WAT browning

a-c Infrared images (**a**), oxygen consumption rate (OCR, **b**), *Ucp1* mRNA (**c**, **left**) and protein levels (**c**, **right**) of BAT from male WT mice i.p. injected with a single dose of PBS (–BK) or 1 mg/kg BK (+BK) before being exposed to 4 °C (Cold) for 90 mins. **d** Whole-body oxygen consumption of male WT mice i.p. injected with a single dose of PBS (–BK) or 1 mg/kg BK (+BK) at 25 °C. **e** The OCR values of Fig. 2e were normalized to non-mito OCR to accurately quantify the changes in the different

components of mitochondrial respiration. **f** mRNA levels of browning marker genes (**f left**) and UCP1 protein levels (**f right**) in sWAT from male WT mice i.p. injected with a single dose of PBS (–BK) or 1 mg/kg BK (+BK) for 30 mins at 25 ℃ (RT). **g** mRNA levels of browning marker genes (**g left**) and UCP1 protein levels (**g right**) in sWAT from male WT mice i.p. injected with a single dose of PBS (–BK) or 1 mg/kg BK (+BK) before being exposed to 4 ℃ (Cold) for 90 mins. The mice in a, b, c, d, f and g were remained at RT or exposed to cold immediately after injection in the absence of food and water. For **a**, n = 4 in each group. For **b**, n = 7 (–BK) and 8 (+BK) mice per group. For **c**, n = 6 (–BK) and 5 (+BK) mice per group (left); n = 5 in each group (right). For **d**, n = 6 in each group. For **e**, n = 5 (–BK) and 6 (+BK) in each group. For **f**, n = 6 (–BK) and 7 (+BK) mice per group (left); n = 6 in each group (right). For **g**, n = 7 (left) and 6 (right) in each group. Mean ± SEM are representative of at least two independent experiments (**a**, **b**, **c**, **d**, **f**, **g**); two-tailed unpaired Student's t-test (**a**, **b**, **c**, **e**, **f**, **g**), two-way RM ANOVA with Geisser-Greenhouse's correction followed by post hoc unpaired t-test (**d**). Source data are provided as a Source data file.

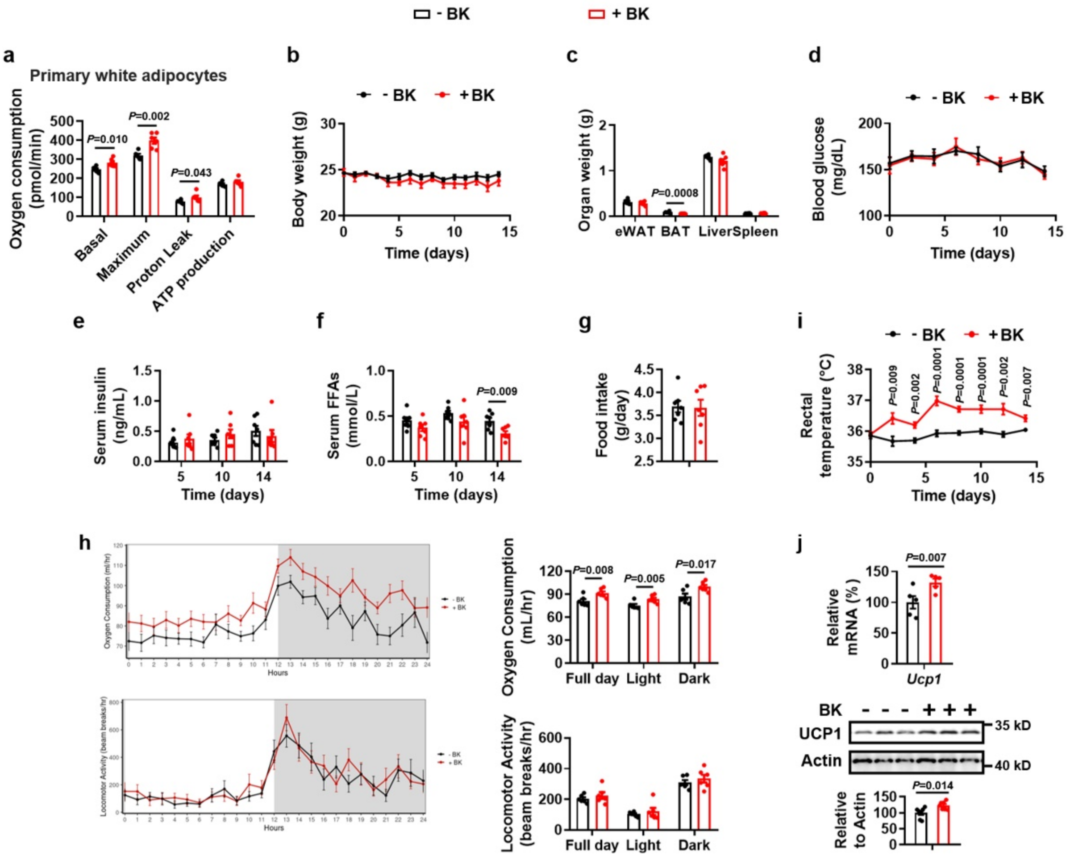

Supplementary Fig. 5 Physiological parameters of mice treated with BK for 14 days

a The oxygen consumption rate (OCR) values of Fig. 3g were normalized to non-mito OCR to accurately quantify the changes in the different components of mitochondrial respiration. **b-j** Body weight (**b**), organ weight (**c**), blood glucose levels (**d**), serum insulin levels (**e**), serum FFAs levels (**f**), food intake (**g**), energy expenditure (**h**), rectal temperature (**i**) and BAT UCP1 expression (**j**) of male WT mice i.p. injected with PBS (–BK) or 1 mg/kg BK (+BK) once a day for 14 days at 25 °C. For **a** and **h**, n = 6 in each group. For **b-g** and **i**, n = 7 in each group. For **j**, n = 5 (up) and 7 (bottom) in each group. Mean ± SEM are representative of at least two independent experiments (**b-j**); two-tailed unpaired Student’s t-test (**a-g**, **i**, **j**); indirect calorimetry

data (**h**) were analyzed using ANCOVA/Generalized Linear Model with body mass as a covariate in CaIR (version 1.3, https://calrapp.org/). Source data are provided as a Source data file.

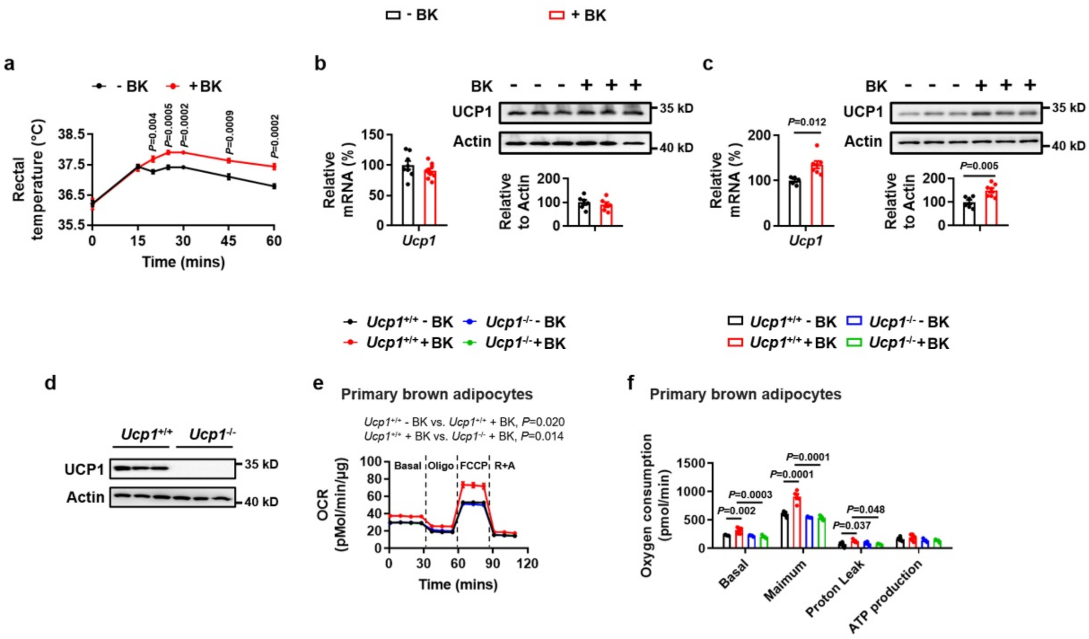

Supplementary Fig. 6 The effects of BK on body temperature and BAT UCP1 expression

a Rectal temperature in male WT mice i.p. injected with a single dose of PBS (–BK) or 1 mg/kg BK (+BK) at 25 °C in the absence of food and water. **b**, **c** *Ucp1* mRNA (**left**) and protein levels (**right**) in BAT of male WT mice at 10 min (**b**) and 15 min (**c**) after i.p. injection of a single dose of PBS (–BK) or 1 mg/kg BK (+BK) at 25 °C in the absence of food and water. **d** UCP1 protein levels in BAT of male control (*Ucp1*$^{+/+}$) or *Ucp1* knockout (*Ucp1*$^{-/-}$) mice. **e**, **f** The oxygen consumption rate (OCR) of primary brown adipocytes from control (*Ucp1*$^{+/+}$) or UCP1 knockout (*Ucp1*$^{-/-}$) mice treated without (–BK) or with (+BK) 0.1 μM BK for 24 hrs; reagents oligomycin (Oligo), carbonyl cyanide 4- (trifluoromethoxy) phenylhydrazone (FCCP), rotenone (R) and

antimycin A (A) were added sequentially as indicated (**e**); the OCR values from **e** were normalized to non-mito OCR to accurately quantify the changes in the different components of mitochondrial respiration (**f**). For **a**, n = 6 per group. For **b**, n=8 (–BK) and 9 (+BK) mice per group (left); n = 6 mice per group (right). For **c**, n = 5 (–BK) and 7 (+BK) mice per group (left); n = 6 (–BK) and 7 (+BK) mice per group (right). For **e** and **f**, n = 5 in each group. Mean ± SEM are representative of at least two independent experiments (**a-c**, **e**, **f**); two-way RM ANOVA with Geisser-Greenhouse's correction followed by post hoc unpaired t-test (**a**), two-tailed unpaired Student's t-test (**b**, **c**), two-way RM ANOVA with Geisser-Greenhouse's correction followed by Tukey's multiple comparisons test (**e**); ordinary two-way ANOVA with Tukey's multiple comparisons test (**f**). Source data are provided as a Source data file.

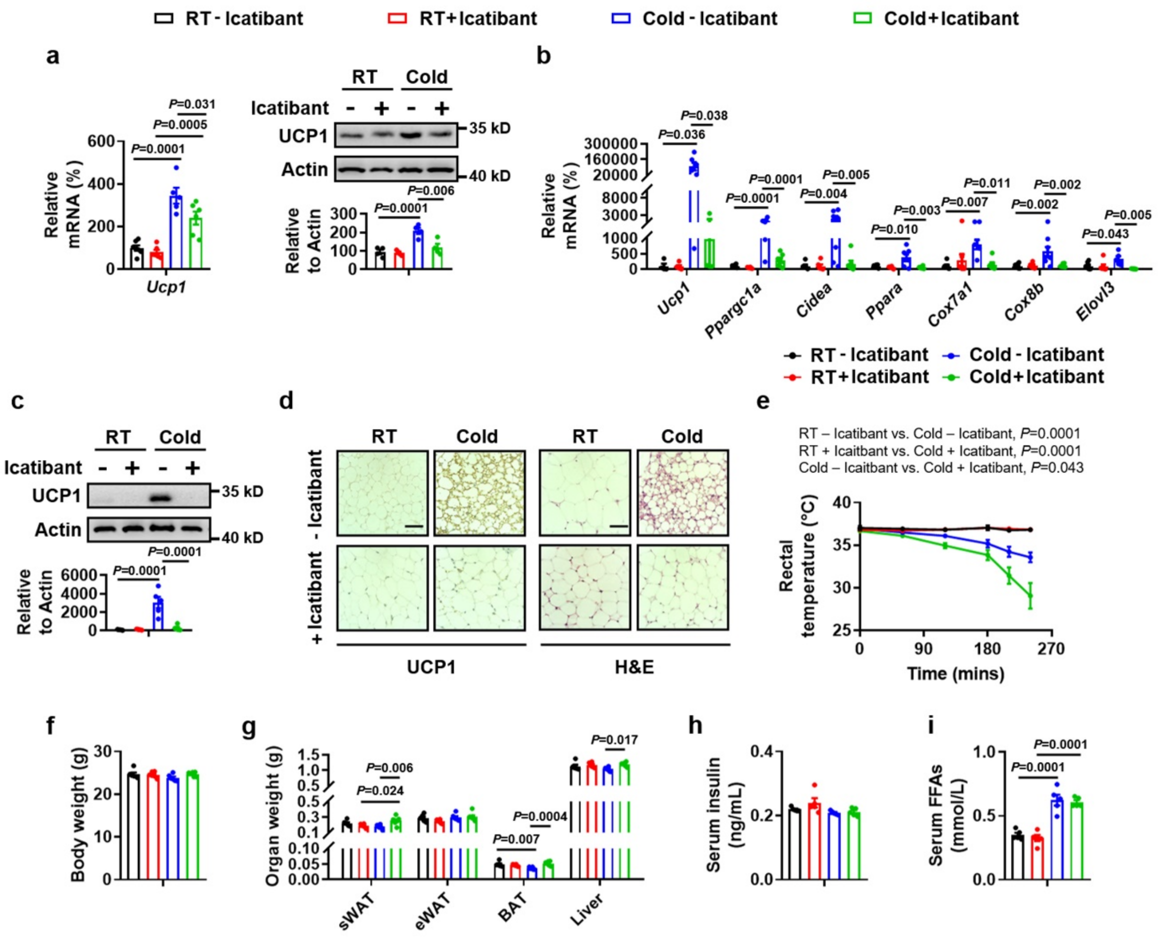

Supplementary Fig. 7 Effects of icatibant on BAT thermogenesis and WAT browning

Male WT mice received one i.p. injection of PBS (–Icatibant) or 1 mg/kg icatibant (+ Icatibant). The mice were immediately exposed to 4 °C (Cold) or maintained at 25 °C (RT) for 3 hrs after injection in the absence of food and water. *Ucp1* mRNA (**a**, **left**) and protein levels (**a**, **right**) in BAT, mRNA levels of browning marker genes (**b**), UCP1 protein levels (**c**), representative immunohistochemistry (IHC) staining of UCP1 (**d**, **left**), representative H&E staining (**d**, **right**) in sWAT, body weight (**f**), organ weight (**g**), serum insulin (**h**) and FFAs (**i**) are shown. Scar bars, 50 μm (**d**). Rectal temperature at different time point was also examined (**e**). For **a**, n = 7 (RT–

Icatibant), 6 (RT+Icatibant, Cold+Icatibant) and 5 (Cold–Icatibant) mice per group (left); n = 4 in each group (right). For **b**, n = 4 or 7 (RT–Icatibant), 5-7 (RT+Icatibant, Cold–Icatibant) and 4-7 (Cold+Icatibant) mice per group. For **c**, n = 5 in each group. For **e**, n = 5 (RT–Icatibant, Cold–Icatibant, Cold+Icatibant) and 7 (RT+Icatibant) mice per group. For **f** and **g**, n = 6 (RT–Icatibant, RT+Icatibant, Cold–Icatibant) and 7 (Cold+Icatibant) mice per group. For **h** and **i**, n = 5 in each group. Mean ± SEM are representative of at least two independent experiments; ordinary two-way ANOVA with Tukey's multiple comparisons test (**a-c**, **f-i**), two-way RM ANOVA with Geisser-Greenhouse's correction followed by Tukey's multiple comparisons test (**e**). Source data are provided as a Source data file.

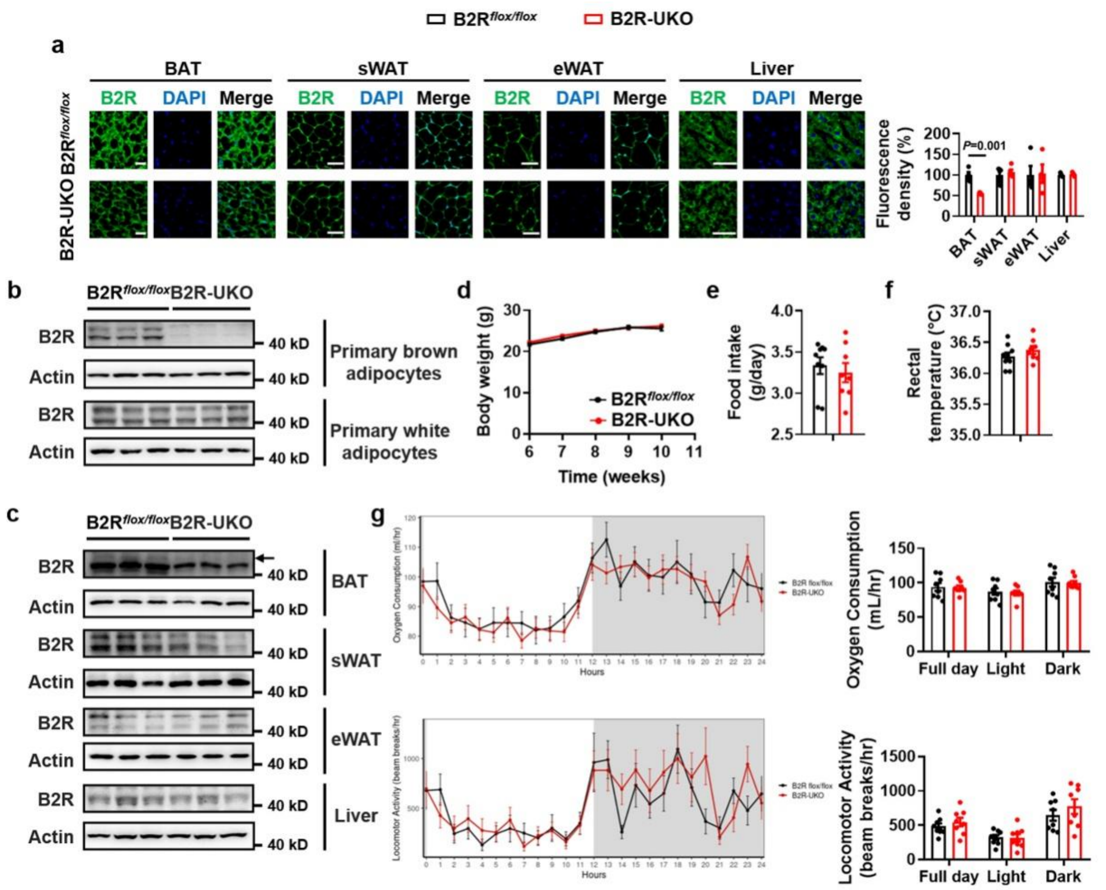

Supplementary Fig. 8 Physiological parameters of B2R-UKO mice

a, **d-g** Representative immunofluorescence (IF) staining of bradykinin receptor B2 (B2R) in different tissues (**a**), body weight (**d**), food intake (**e**), rectal temperature(**f**) and energy expenditure (**g**) at 25 °C from male B2R*flox/flox* or B2R-UKO mice. Scar bars, 20 (BAT) or 50 μm (sWAT, eWAT and liver). **b** B2R protein levels in primary brown and white adipocytes of male B2R*flox/flox* and B2R-UKO mice. **c** B2R protein levels in different tissues of B2R*flox/flox* and B2R-UKO mice at 4 °C for 6 hrs. For **a**, n = 4 (B2R*flox/flox* BAT, eWAT; B2R-UKO BAT, eWAT) or 5 (B2R*flox/flox* sWAT, Liver; B2R-UKO sWAT, Liver) mice per group. For **d**, n = 9 in each group. For **e** and **f**, n = 9 (B2R*flox/flox*) and 8 (B2R-UKO) in each group. For **g**, n = 8 in each group. Mean ± SEM are representative of at least two independent experiments (**a-g**); two-tailed

unpaired Student's t-test (**a**, **d**, **e**, **f**); indirect calorimetry data (**g**) were analyzed using ANCOVA/Generalized Linear Model with body mass as a covariate in CaIR (version 1.3, https://calrapp.org/). Source data are provided as a Source data file.

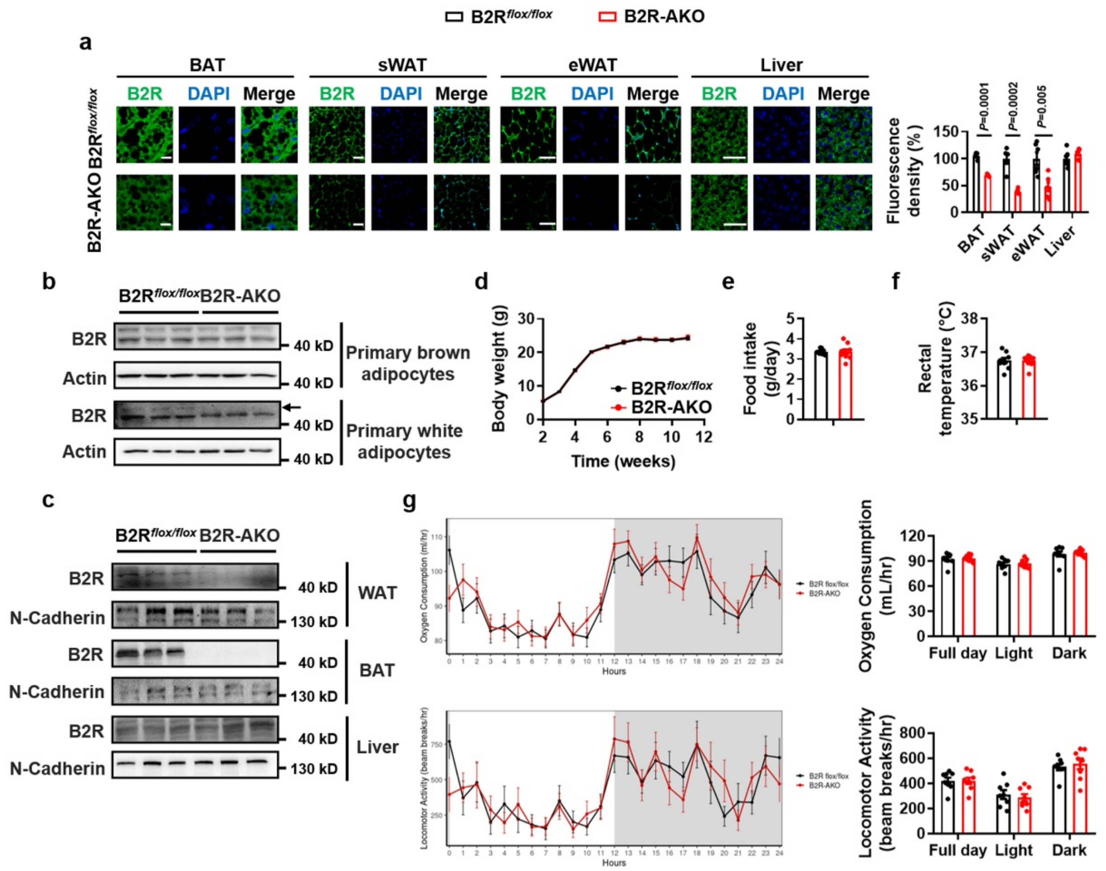

Supplementary Fig. 9 Physiological parameters of B2R-AKO mice

a, **c-g** Representative immunofluorescence (IF) staining of bradykinin receptor B2 (B2R) in different tissues (**a**), B2R levels of membrane proteins extracted from different tissues (**c**), body weight (**d**), food intake (**e**), rectal temperature(**f**) and energy expenditure (**g**) at 25 °C from male B2R*flox/flox* or B2R-AKO mice. Scar bars, 20 (BAT) or 50 μm (sWAT, eWAT and liver). **b** B2R protein levels in primary brown and white adipocytes of male B2R*flox/flox* and B2R-AKO mice. For **a**, n = 5 (BAT, sWAT), 6

(eWAT) and 8 (Liver) mice per group. For **d**, n = 3 (2 weeks) and 6 (3-11 weeks) in B2R*flox/flox* group; n = 4 (2 weeks) and 5 (3-11 weeks) in B2R-AKO group. For **e** and **f**, n = 9 in each group. For **g**, n = 8 in each group. Mean ± SEM are representative of at least two independent experiments (**a-g**); two-tailed unpaired Student's t-test (**a**, **d**, **e**, **f**), indirect calorimetry data (**g**) were analyzed using ANCOVA/Generalized Linear Model with body mass as a covariate in CaIR (version 1.3, https://calrapp.org/). Source data are provided as a Source data file.

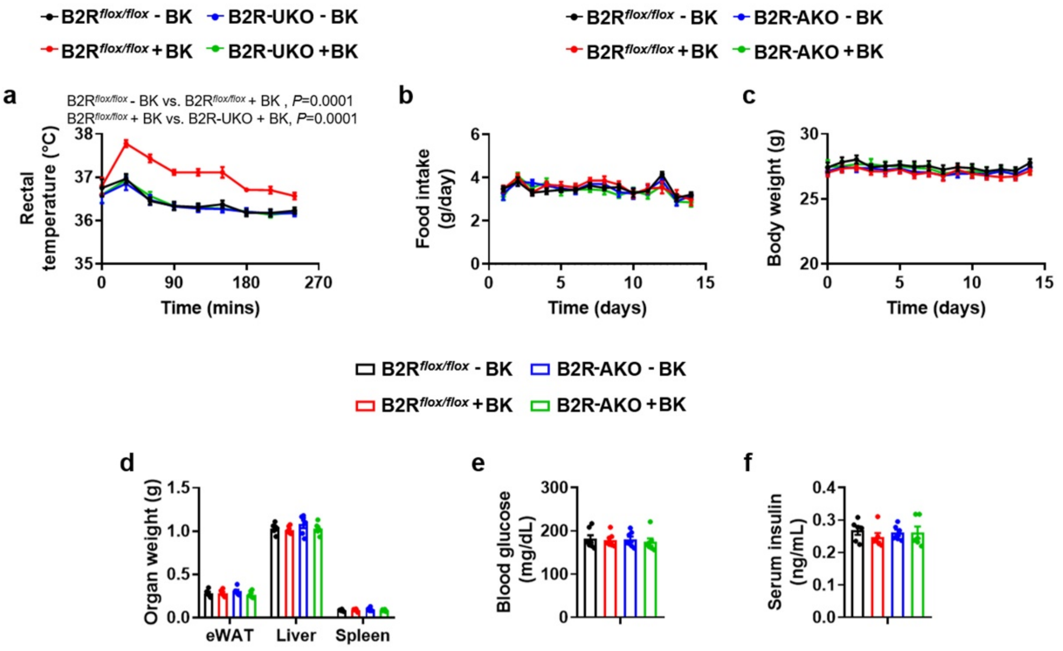

Supplementary Fig. 10 Physiological parameters of B2R-UKO and B2R-AKO mice treated with BK

a Rectal temperature of B2R*flox/flox* or B2R-UKO male mice i.p. injected with a single dose of PBS (–BK) or 1 mg/kg BK (+BK) for different amounts of time at 25 °C in the absence of food and water. **b-f** Food intake (**b**), body weight (**c**), organ weight (**d**), blood glucose levels (**e**), and serum insulin levels (**f**) of male B2R*flox/flox* and

B2R-AKO mice i.p. injected with PBS (–BK) or 1 mg/kg BK (+BK) once a day for 14 days at 25 °C. For **a**, n = 8 (B2R$^{flox/flox}$–BK) and 9 (B2R$^{flox/flox}$+BK, B2R-UKO–BK, B2R-UKO+BK) mice per group. For **b**, **c** and **e**, n = 7 in each group. For **d** and **f**, n = 6 in each group. Mean ± SEM are representative of at least two independent experiments (**a-f**). Two-way RM ANOVA with Geisser-Greenhouse's correction followed by Tukey's multiple comparisons test (**a**), ordinary two-way ANOVA with Tukey's multiple comparisons test (**b-f**). Source data are provided as a Source data file.

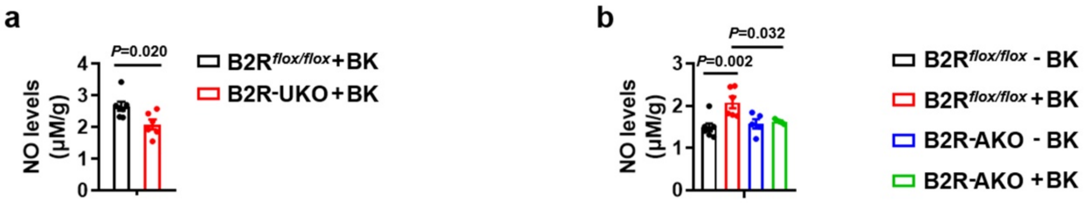

Supplementary Fig. 11 Nitric oxide (NO) levels in adipose tissue of B2R-UKO and B2R-AKO mice

a NO levels of BAT from B2R$^{flox/flox}$ or B2R-UKO male mice i.p. injected with a single dose of 1 mg/kg BK after 30 mins at 25 °C in the absence of food and water. **b** NO levels of sWAT from B2R$^{flox/flox}$ or B2R-AKO male mice i.p. injected with PBS (–BK) or 1 mg/kg BK (+BK) once a day for 14 days at 25 °C. For **a**, n = 7 (B2R$^{flox/flox}$+BK) and 6 (B2R-UKO+BK) mice per group. For **b**, n = 7 (B2R$^{flox/flox}$–BK), 6 (B2R$^{flox/flox}$+BK) and 5 (B2R-AKO–BK, B2R-AKO+BK) mice per group. Mean ± SEM are representative of at least two independent experiments (**a**, **b**); two-tailed unpaired Student's t-test (**a**), ordinary two-way ANOVA with Tukey's multiple

comparisons test (**b**). Source data are provided as a Source data file.

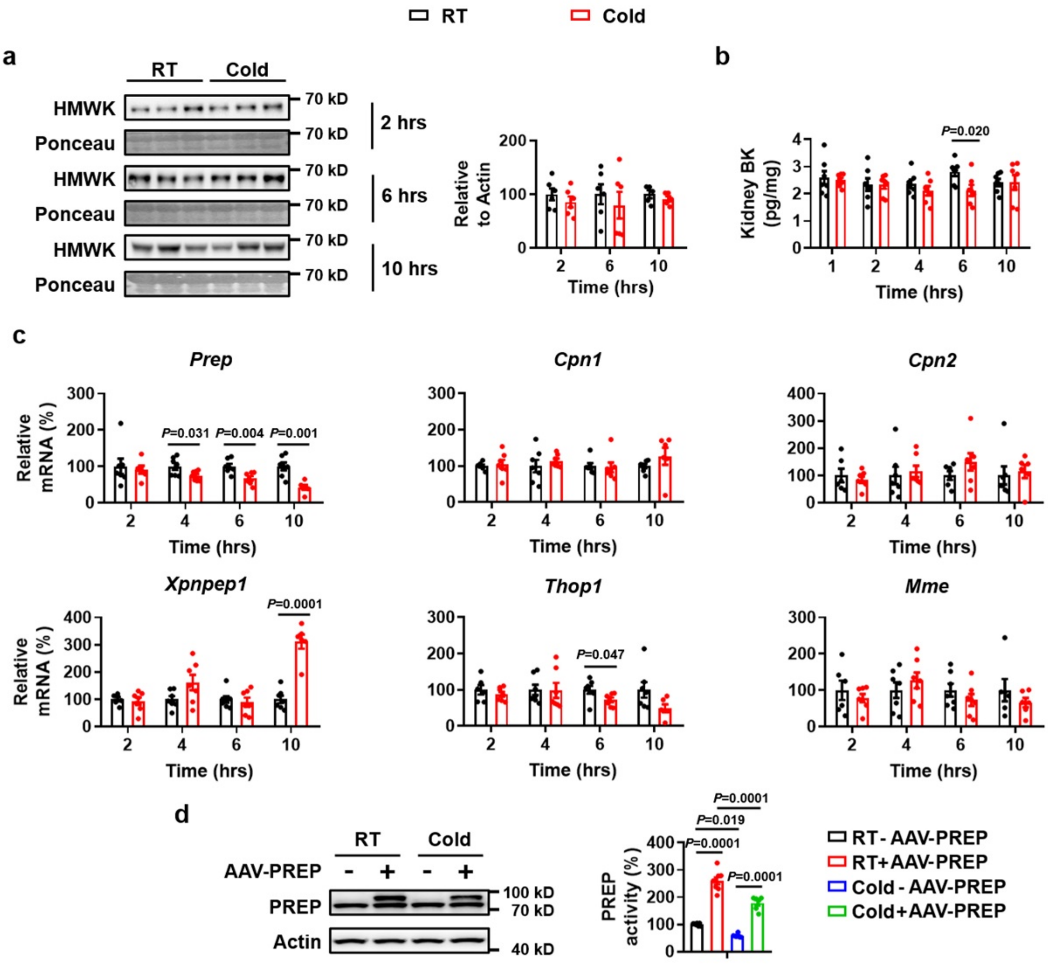

Supplementary Fig. 12 Effects of acute cold exposure on serum HMWK levels, kidney BK content and hepatic BK-degrading enzymes expression

a-c Serum HMWK levels (**a**), kidney BK content (**b**) and hepatic BK-degrading enzymes mRNA levels (**c**) from male WT mice exposed to 25 °C (RT) or 4 °C (Cold) for different amounts of time in the absence of food and water. **d** Hepatic PREP protein levels (left) and activity (right) from WT male mice infected with (+AAV-PREP) or without (–AAV-PREP) AAV-PREP and exposed to 25 °C (RT) or 4 °C (Cold) for 2 hrs in the absence of food and water. For **a**, n = 6 (RT 2hr, 6hrs;

Cold 2hrs, 6hrs) and 5 (RT 10hrs; Cold 10hrs) mice per group; For **b**, n = 7 in each group. For **c**, n = 6 or 7 in each group. For **d**, n = 7 (RT–AAV-PREP), 8 (RT+AAV-PREP) and 6 (Cold– AAV-PREP, Cold+AAV-PREP) mice per group. Mean ± SEM are representative of at least two independent experiments (**a-d**); two-tailed unpaired Student's t-test (**a-c**), ordinary two-way ANOVA with Tukey's multiple comparisons test (**d**). Source data are provided as a Source data file.

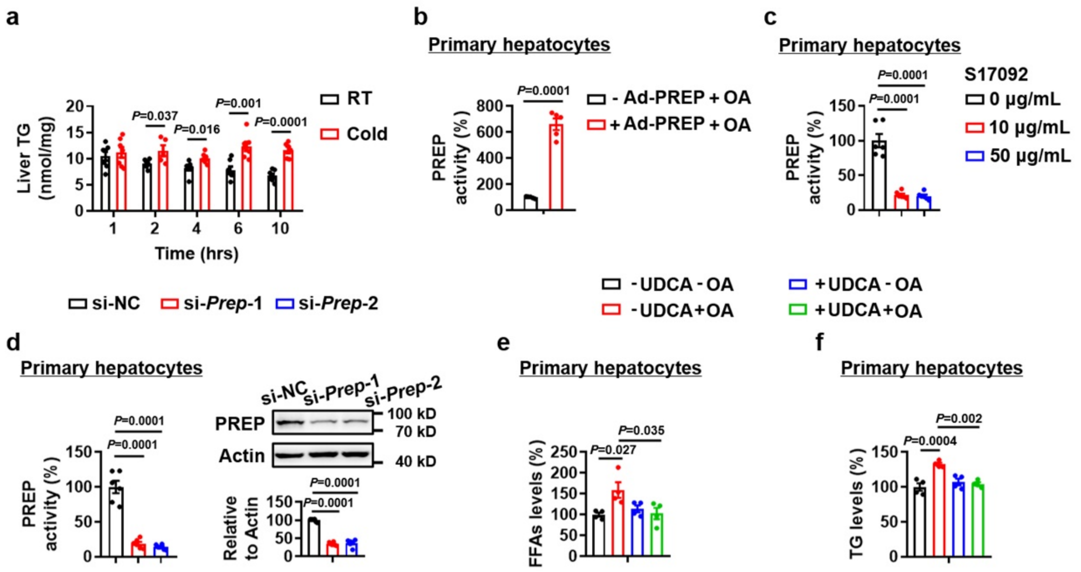

Supplementary Fig. 13 PREP activity in primary hepatocytes under different treatments

a Triglyceride (TG) levels in the liver of male WT mice exposed to 25 °C (RT) or 4 °C (Cold) for different amounts of time in the absence of food and water. **b** PREP activity in primary hepatocytes infected with (+Ad-PREP) or without (–Ad-PREP) Ad-PREP in the presence (+OA) of 1 mM oleic acid (OA) for 6 hrs. **c** PREP activity in primary hepatocytes treated with 0, 10 or 50 μg/mL S17092 for 6 hrs. **d** PREP activity and protein levels in primary hepatocytes transfected with (si-*Prep*) or

without (si-NC) *Prep* siRNA for 72 hrs. **e**, **f** Free fatty acids (FFAs, **e**) and TG (**f**) levels in primary hepatocytes treated with (+UDCA) or without (–UDCA) 0.1 mM ursodeoxycholic acid (UDCA) in the absence (–OA) or presence (+OA) of 1 mM OA for 6 hrs. For **a**, n = 7 (RT 1hr, 4hrs, 6hrs, 10hrs; Cold 1hr, 6hrs), 6 (RT 2hrs; Cold 4hrs, 10hrs) and 5 (Cold 2hrs) mice per group. For **b**, n = 5 in each group. For **c** and **d**, n = 6 in each group. For **e** and **f**, n = 4 in each group. Mean ± SEM are representative of at least two independent experiments (**a**) or at least three independent experiments (**b-f**); two-tailed unpaired Student's t-test (**a**, **b**), ordinary one-way ANOVA with Dunnett's test (**c**, **d**), ordinary two-way ANOVA with Tukey's multiple comparisons test (**e**, **f**). Source data are provided as a Source data file.

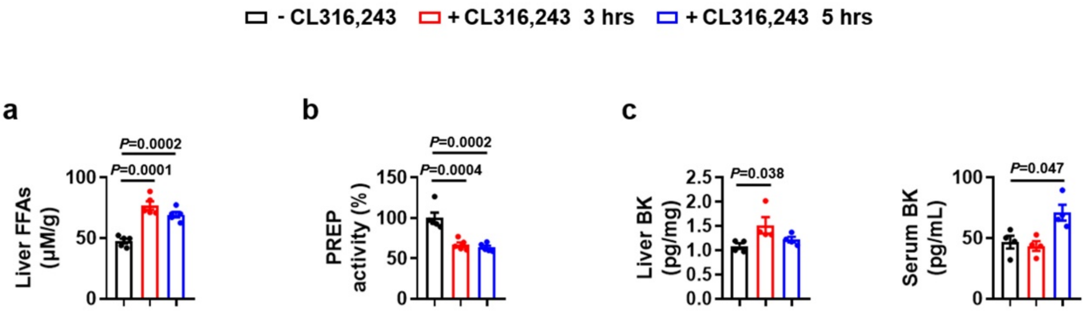

Supplementary Fig. 14 Effects of CL316,243 on PREP activity and BK levels

Male WT mice were i.p. injected with a single dose of normal saline (–CL316,243) or 1 mg/kg CL316,243 (+CL316,243) for 3 or 5 hrs in the absence of food and water at 25 °C. **a** Free fatty acids (FFAs) levels in the liver. **b** Hepatic PREP activity. **c** Hepatic and serum BK levels. For **a** and **b**, n = 5 in each group. For **c**, n = 4 in each group. Mean ± SEM are representative of at least two independent experiments (**a-c**); ordinary one-way ANOVA with Dunnett's test (**a-c**). Source data are provided as a

Source data file.

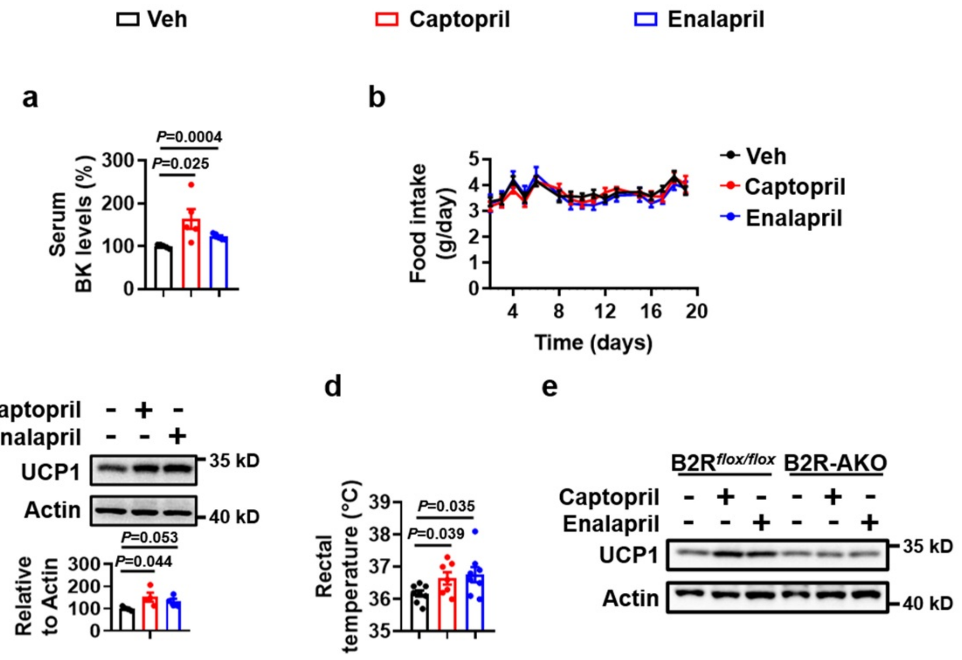

Supplementary Fig. 15 Effects of ACEIs on food intake and BAT UCP1 expression

a-d Serum BK levels (**a**), food intake (**b**), BAT UCP1 protein levels (**c**), and rectal temperature (**d**) of male WT mice treated with 40 mg/kg captopril or 70 mg/kg enalapril by gavage once a day for 21 days at 25 °C. BK levels were measured by LC-MS/MS (**a**). **e** UCP1 protein levels in BAT of B2R$^{flox/flox}$ or B2R-AKO male mice treated with 40 mg/kg captopril or 70 mg/kg enalapril by gavage once a day for 21 days at 25 °C. For **a**, n = 5 in each group. For **b**, n = 8 in each group. For **c**, n = 4 in each group. For **d**, n = 8 (Veh, Enalapril) and 7 (Captopril) mice per group. Mean ± SEM are representative of at least two independent experiments (**a-d**); two-tailed unpaired Student's t-test (**a-d**). Source data are provided as a Source data file.

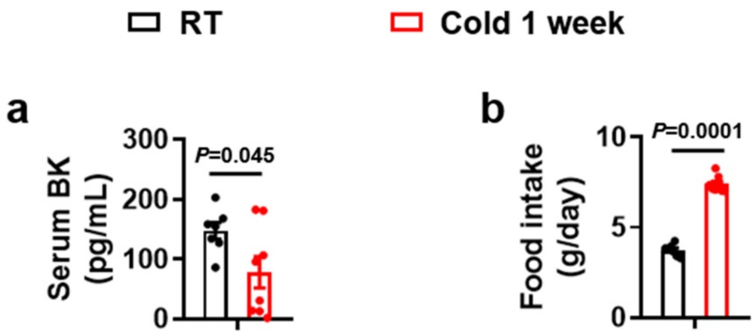

Supplementary Fig. 16 Impact of chronic cold on circulating BK levels

Serum BK levels (**a**) and food intake (**b**) of male WT mice exposed to 25 °C (RT) or 4 °C (Cold) for 1 week in the presence of food and water. For **a** and **b**, n = 7 in RT group and n = 8 in Cold group. Mean ± SEM are representative of at least two independent experiments (**a**, **b**); two-tailed unpaired Student's t-test. Source data are provided as a Source data file.

Supplementary Table 1. List of oligonucleotide primer pairs used in RT-qPCR analysis

Target Gene	Forward primer (5' – 3')	Reverse primer (5' – 3')
Cidea	TGCTCTTCTGTATCGCCCAGT	GCCGTGTTAAGGAATCTGCTG
Cox7a1	CAGCGTCATGGTCAGTCTGT	AGAAAACCGTGTGGCAGAGA
Cox8b	GTTCCAGCAGGATGGGTCTTAG	TTCATGCTGCGGAGCTCTT
Cpn1	TCCAAGTTTGTTACCCCGGTG	CTGCGCCCGATGTTGTAGAG
Cpn2	GTGCTGGGTCTCACTCCTG	GTTGGGGCTACCGCTGAAA
Elovl3	GGCACCATCTTTGGCATACTG	CGTTGTTGTGTGGCATCCTT
Gapdh	TGTGTCCGTCGTGGATCTGA	CCTGCTTCACCACCTTCTTGAT
Mme	CTCTCTGTGCTTGTCTTGCTC	GACGTTGCGTTTCAACCAGC
Ppara	TCTGTGGGCTCACTGTTCTG	AACTACCTGCTCAGGGCTCA
Ppargc1a	GATGGCACGCAGCCCTAT	CTCGACACGGAGAGTTAAAGGAA

Prep	ACCTCCGTGCAGGAGTATCAT	TCTGGGTCTTCAAGCCAAGAATA
Thop1	CAGTGCAGAGGAACATTCTCG	CACGTCCTGTCTCATGCTCAT
Ucp1	ACTGCCACACCTCCAGTCATT	CTTTGCCTCACTCAGGATTGG
Xpnpep1	CGACAAGCTATGAGGAACTCCG	GTCACAGGGTGCAATGTACTC
