# Supplementary material for: Reduced hepatic bradykinin degradation accounts for cold-induced BAT thermogenesis and WAT browning in male mice
Source: Nat Commun. 2023 May 2;14:2523. doi: 10.1038/s41467-023-38141-0 (PMC10154316; doi:10.1038/s41467-023-38141-0)
Supplement: Supplementary file 2 — Description of Additional Supplementary Files [file 41467_2023_38141_MOESM2_ESM.pdf]

Description of Additional Supplementary Files

File Name: Supplementary Data 1
Description: RNA-sequencing analysis of BAT from male WT mice i.p. injected with a single dose of PBS (–BK) or 1 mg/kg BK (+BK) for 30 mins in the absence of food and water at 25 ℃. The screening criteria for differentially expressed genes were as follows: log2|FC|≥1, Q value≤0.05. n=4 per group.
