# Supplementary material for: Reduced hepatic bradykinin degradation accounts for cold-induced BAT thermogenesis and WAT browning in male mice
Source: Nat Commun. 2023 May 2;14:2523. doi: 10.1038/s41467-023-38141-0 (PMC10154316; doi:10.1038/s41467-023-38141-0)
Supplement: Supplementary file 4 — Reporting Summary [file 41467_2023_38141_MOESM4_ESM.pdf]

nature portfolio

Corresponding author(s): Feifan Guo

Last updated by author(s): Mar 30, 2023

Reporting Summary

Nature Portfolio wishes to improve the reproducibility of the work that we publish. This form provides structure for consistency and transparency in reporting. For further information on Nature Portfolio policies, see our Editorial Policies and the Editorial Policy Checklist.

Statistics

For all statistical analyses, confirm that the following items are present in the figure legend, table legend, main text, or Methods section.

n/a	Confirmed	
☐	☒	The exact sample size (*n*) for each experimental group/condition, given as a discrete number and unit of measurement
☐	☒	A statement on whether measurements were taken from distinct samples or whether the same sample was measured repeatedly
☐	☒	The statistical test(s) used AND whether they are one- or two-sided<br>*Only common tests should be described solely by name; describe more complex techniques in the Methods section.*
☒	☐	A description of all covariates tested
☐	☒	A description of any assumptions or corrections, such as tests of normality and adjustment for multiple comparisons
☐	☒	A full description of the statistical parameters including central tendency (e.g. means) or other basic estimates (e.g. regression coefficient) AND variation (e.g. standard deviation) or associated estimates of uncertainty (e.g. confidence intervals)
☐	☒	For null hypothesis testing, the test statistic (e.g. *F*, *t*, *r*) with confidence intervals, effect sizes, degrees of freedom and *P* value noted<br>*Give P values as exact values whenever suitable.*
☒	☐	For Bayesian analysis, information on the choice of priors and Markov chain Monte Carlo settings
☒	☐	For hierarchical and complex designs, identification of the appropriate level for tests and full reporting of outcomes
☒	☐	Estimates of effect sizes (e.g. Cohen's *d*, Pearson's *r*), indicating how they were calculated

Our web collection on statistics for biologists contains articles on many of the points above.

Software and code

Policy information about availability of computer code

Data collection

1. Rectal temperature: Digital thermometer (Physitemap Instruments)
2. Infrared image: Infrared camera (Magnity Electronics)
3. Plasma, tissue, cell parameters and enzyme activity: Microplate reader (PerkinElmer)
4. Mitochondrial function and respiration: Seahorse XF24 Extracellular Flux Analyzer (Seahorse Bioscience)
5. Measurement of [Ca2+]: FLIPR Tetra (Molecular Devices)
6. Body composition: Magnetic resonance imaging (EchoMRI)
7. Indirect calorimetry: Metabolic cage (Columbus Instruments)
8. Histological analysis: Confocal microscope (Zeiss) or PerkinElmer Vectra platform
9. RT-qPCR: ABI QuantStudio™ 6 Flex Real-Time PCR System
11. Immunoblotting: Biorad
12. RNA-seq: MGI 2000

Data analysis

1. Fluorescence density: FIJI-Image J (version 1.53c)
2. Immunoblotting: Tanon Gis
3. Mitochondrial function and respiration: Seahorse Wave Desktop Software (Agilent, 2.6)
4. Indirect calorimetry: CalR version 1.3 (https://calrapp.org/)
5. Infrared image data: ThermoX 2.5.3.4
6. Statistical analysis: GraphPad Prsim 8
7. RNA-seq: The sequencing data was filtered with SOAPnuke by (1) Removing reads containing sequencing adapter; (2) Removing reads whose low-quality base ratio (base quality less than or equal to 15) is more than 20%; (3) Removing reads whose unknown base ('N' base) ratio is more than 5%, afterwards clean reads were obtained and stored in FASTQ format. The subsequent analysis and data mining were

performed on Dr. Tom Multi-omics Data mining system (https://biosys.bgi.com). HISAT2 (v2.1.0) was applied to align the clean reads to the gene set, in which known and novel, coding and noncoding transcripts were included. The heatmap was drawn by pheatmap (v1.0.8) according to the gene expression difference in different samples. Essentially, differential expression analysis was performed using the DEGseq with Q value ≤ 0.05.

For manuscripts utilizing custom algorithms or software that are central to the research but not yet described in published literature, software must be made available to editors and reviewers. We strongly encourage code deposition in a community repository (e.g. GitHub). See the Nature Portfolio guidelines for submitting code & software for further information.

Data

Policy information about availability of data

All manuscripts must include a data availability statement. This statement should provide the following information, where applicable:
- Accession codes, unique identifiers, or web links for publicly available datasets
- A description of any restrictions on data availability
- For clinical datasets or third party data, please ensure that the statement adheres to our policy

All data presented in this study are available within the Figures and its supplementary information file. Source data including uncropped western blots and raw microscopy images are provided with this manuscript. The high-throughput sequencing data for this study have been deposited in National Center for Biotechnology Information. The accession number for the RNA-seq data reported in this manuscript is PRJNA948923.

Human research participants

Policy information about studies involving human research participants and Sex and Gender in Research.

Reporting on sex and gender	N/A
Population characteristics	N/A
Recruitment	N/A
Ethics oversight	N/A

Note that full information on the approval of the study protocol must also be provided in the manuscript.

Field-specific reporting

Please select the one below that is the best fit for your research. If you are not sure, read the appropriate sections before making your selection.

☒ Life sciences ☐ Behavioural & social sciences ☐ Ecological, evolutionary & environmental sciences

For a reference copy of the document with all sections, see nature.com/documents/nr-reporting-summary-flat.pdf

Life sciences study design

All studies must disclose on these points even when the disclosure is negative.

Sample size	No statistical methods were used to predetermine sample size. For animals studies, in order to minimize any potential bias, we assigned mice randomly of the same genotype to different treatments. At least 4 mice per group at each time point were operated to ensure adequate sample size. All experiments were taken from at least 4 samples (biological replicates) with similar results.
Data exclusions	No data was excluded from the analyses.
Replication	All attempts of replication were successful. All experimental data were reproduced in multiple independent experiments as indicated in the figure legends.
Randomization	Mice were allocated randomly. In experiments involving WT and KO animals, mice were allocated to each group based on genotype. All mice used in the manuscript were age and sex matched.
Blinding	Experiments performance was not completely blinded because some operations last a few hours. In order to minimize the effect of biorhythms on mice, the control group and the experimental group mice were alternately performed. Each experiment was associated with proper control, and samples were collected and analyzed under the same conditions.

Reporting for specific materials, systems and methods

We require information from authors about some types of materials, experimental systems and methods used in many studies. Here, indicate whether each material, system or method listed is relevant to your study. If you are not sure if a list item applies to your research, read the appropriate section before selecting a response.

Materials & experimental systems

n/a	Involved in the study
☐	☒ Antibodies
☒	☐ Eukaryotic cell lines
☒	☐ Palaeontology and archaeology
☐	☒ Animals and other organisms
☒	☐ Clinical data
☒	☐ Dual use research of concern

Methods

n/a	Involved in the study
☒	☐ ChIP-seq
☒	☐ Flow cytometry
☒	☐ MRI-based neuroimaging

Antibodies

Antibodies used: anti-UCP1 (1:500 for IHC, 1:1000 for WAT, 1:10000 for BAT; Abcam; ab10983, ab209483); anti-BDKRB2 (1:200 for IHC, 1:1000 for WB; ABclonal; A2844); anti-PREP (1:1000; A9838; ABclonal); anti-HMWK (1:500; sc-23914; Santa Cruz); anti-β-Actin (1:3000; 66009-1-Ig; Proteintech); anti-N-Cadherin (1:1000; A19083; ABclona); Donkey anti-Rabbit IgG Alexa Fluor™ 488 (1:1000; A-21206; Thermo Fisher)

Validation: All antibodies used in this paper were validated by WB/IHC/IF in published articles, or in reference to manufacturer's websites, or in relevant knockout mice.
anti-UCP1 (ab10983): Applications: WB, IHC. Reacts with: Mouse, Rat
anti-UCP1 (ab209483): Applications: WB, IHC, IP. Reacts with: Mouse, Rat
anti-BDKRB2: Applications: WB, IHC. Reacts with: Human, Mouse, Rat
anti-PREP: Applications: WB. Reacts with: Human, Mouse
anti-HMWK: Applications: WB, IP, IHC, IF. Reacts with: Human, Mouse, Rat
anti-β-Actin: Applications: WB, IP, IHC, IF, FC, CoIP, ChIP, ELISA. Reacts with: Human, Mouse, Rat, Hamster, Zebrafish, Monkey, Dog
anti-N-Cadherin: Applications: WB, IHC, IF. Reacts with: Human, Mouse, Rat
Donkey anti-Rabbit IgG Alexa Fluor™ 488: Applications: IHC, ICC/IF, Flow. Reacts with: Rabbit

Animals and other research organisms

Policy information about studies involving animals; ARRIVE guidelines recommended for reporting animal research, and Sex and Gender in Research

Laboratory animals: Eight- to ten-week-old male C57BL/6J mice were used. Wild type (WT), Bdkrb2flox/flox, Adipoq-Cre, and aged mice were purchased from Shanghai Model Organisms. The UCP1-KO mice were a kind gift from Prof. Xinran Ma (East China Normal University) and the Ucp1-Cre mice were a kind gift from Prof. Jiqiu Wang (Ruijin Hospital, Shanghai Jiaotong University School of Medicine). All mice experiments were performed in accordance with the procedures of the Institutional Animal Care and Use Committee at Fudan University or the Shanghai Institute of Nutrition and Health, Chinese Academy of Sciences. Mice were housed on a 12 hrs light/dark cycle from 7 A.M. to 7 P.M. at room temperature (25°C) with humidity at 40 %-70 % and provided free access to standard chow rodent diets and water. Mice were sacrificed by CO2 inhalation.

Wild animals: The study did not involve wild animals.

Reporting on sex: All mice used in this study were male.

Field-collected samples: The study did not involve samples collected from the field.

Ethics oversight: All mice experiments were performed in accordance with the procedures of the Institutional Animal Care and Use Committee at Fudan University and the Shanghai Institute of Nutrition and Health, Chinese Academy of Sciences.

Note that full information on the approval of the study protocol must also be provided in the manuscript.
